# Taxonomic Composition and Trophic Structure of the Continental Bony Fish Assemblage from the Early Late Cretaceous of Southeastern Morocco

**DOI:** 10.1371/journal.pone.0125786

**Published:** 2015-05-27

**Authors:** Lionel Cavin, Larbi Boudad, Haiyan Tong, Emilie Läng, Jérôme Tabouelle, Romain Vullo

**Affiliations:** 1 Département de géologie et paléontologie, Muséum d’histoire naturelle de Genève, CP 6434, 1211, Geneva 6, Switzerland; 2 Université Moulay Ismail, Faculté des Sciences, Département de Géologie BP. 11201, Zitoune, 50070, Meknès, Morocco; 3 Palaeontological Research and Education Centre, Mahasarakham University, Kantarawichai, Mahasarakham, 44150, Thailand; 4 Fabrique des Savoirs, LA CREA 7 cours Gambetta, Elbeuf, 76500, France; 5 Laboratoire Géosciences Rennes, UMR CNRS 6118, Université de Rennes 1, 263 avenue du Général Leclerc, 35042, Rennes, France; University of Oxford, UNITED KINGDOM

## Abstract

The mid-Cretaceous vertebrate assemblage from south-eastern Morocco is one of the most diversified continental vertebrate assemblages of this time worldwide. The bony fish component (coelacanths, lungfishes and ray-finned fishes) is represented by relatively complete specimens and, mostly, by fragmentary elements scattered along 250 kilometres of outcrops. Here we revisit the bony fish assemblage by studying both isolated remains collected during several fieldtrips and more complete material kept in public collections. The assemblage comprises several lungfish taxa, with the first mention of the occurrence of *Arganodus tiguidiensis*, and possibly two mawsoniid coelacanths. A large bichir cf. *Bawitius*, is recorded and corresponds to cranial elements initially referred to ‘*Stromerichthys*’ from coeval deposits in Egypt. The ginglymodians were diversified with a large ‘*Lepidotes*’ plus two obaichthyids and a gar. We confirm here that this gar belongs to a genus distinctive from Recent gars, contrary to what was suggested recently. Teleosteans comprise a poorly known ichthyodectiform, a notopterid, a probable osteoglossomorph and a large tselfatiiform, whose cranial anatomy is detailed. The body size and trophic level for each taxon are estimated on the basis of comparison with extant closely related taxa. We plotted the average body size versus average trophic level for the Kem Kem assemblage, together with extant marine and freshwater assemblages. The Kem Kem assemblage is characterized by taxa of proportionally large body size, and by a higher average trophic level than the trophic level of the extant compared freshwater ecosystems, but lower than for the extant marine ecosystems. These results should be regarded with caution because they rest on a reconstructed assemblage known mostly by fragmentary remains. They reinforce, however, the ecological oddities already noticed for this mid-Cretaceous vertebrate ecosystem in North Africa.

## Introduction

**Fig 1:**
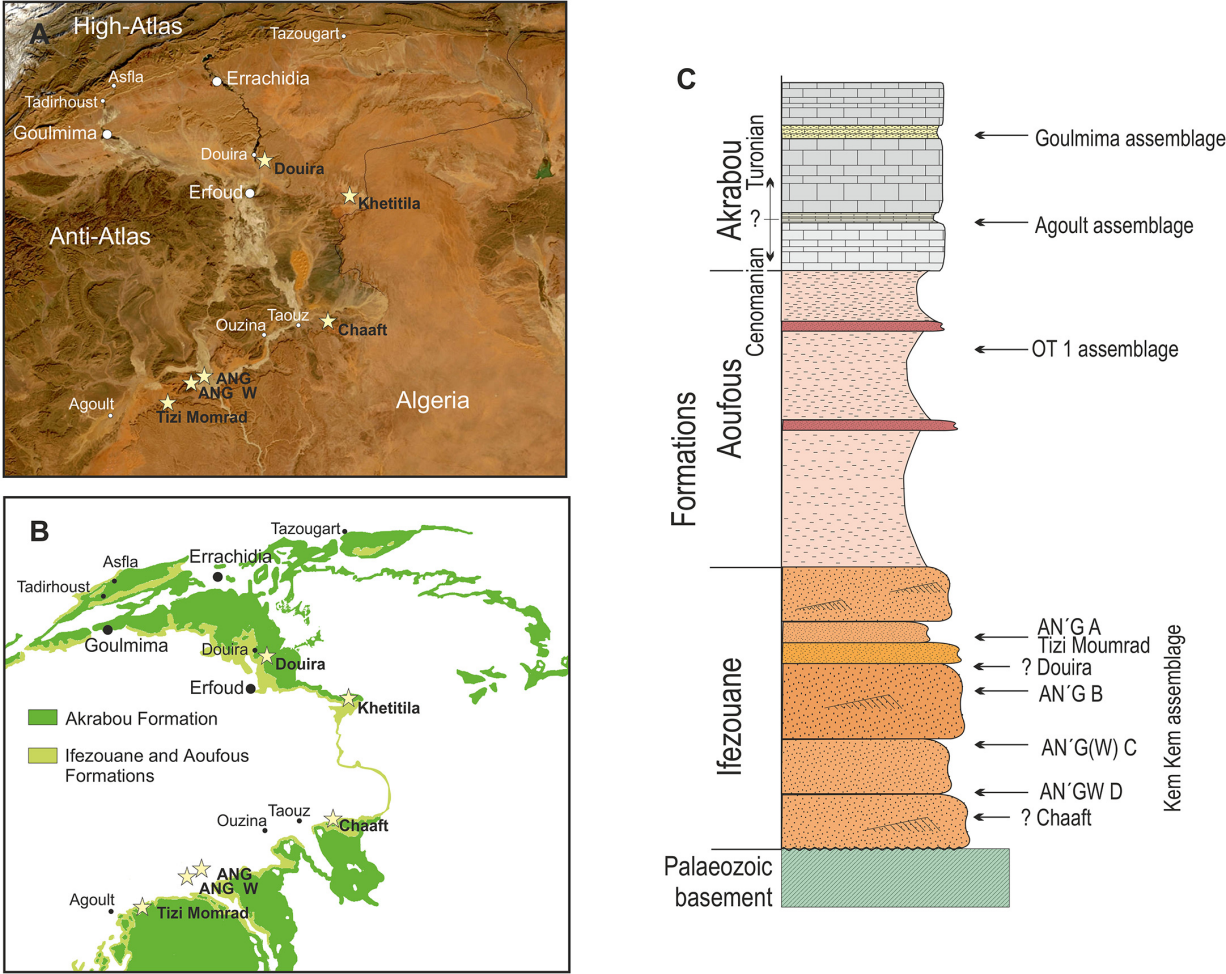
Aerial photograph (A) and geological simplified map (B) of the studied area. The localities mentioned in the text are in black font and the towns and cities are in white font in A. C, Simplified stratigraphic section with the approximate position of the localities. Only the sites situated in the Ifezouane Formation are discussed in the text.

The continental or brackish deposits from the early Cenomanian of the Ifezouane Formation, sometimes gathered with the Aoufous Formation within the Kem Kem beds, have yielded a very rich assemblage including terrestrial, aquatic and aerial vertebrates [1]. The fossils are located in outcrops extending along more than 250 kilometres, which marks the landscape as the basement of a cliff over hanged by a calcareous plateau (hamada), which is formed by the limestone Akrabou Formation. The Ifezouane Formation is composed mainly of detritic sandstone characterised by cross-stratified structures and channel structures filled with microconglomerates. The Aoufous Formation is composed mainly of clayey sandstones and green marls with gypsum and contains few fossils, except in the Djbel Oum Tkout locality (also called OT1 assemblage [2]), whose assemblage is not discussed in this paper. The overhanging Akrabou Formation is a calcareous series corresponding to the settlement of marine environment. The shallow marine fish and invertebrate assemblage from Agoult is located at the base of the Formation, in an area that has also yielded vertebrate remains in the underlying Ifezouane Formation (Gara Sbâa). The Goulmima assemblage is located higher in the stratigraphical series and is situated in the northern part of the basin. The Cenomanian-Turonian boundary is located in the lower part of the Akrabou Formation. The Goulmima assemblage is well dated to the early Turonian on the basis of ammonites, but the Agoult assemblage is regarded as either Late Cenomanian or Early Turonian. Fish taxa from both marine localities are not discussed here, except for a comparison of trophic webs of the Goulmima assemblage with the Kem Kem assemblage (see below). The geographical distribution and the approximate stratigraphic location of these assemblages are presented in Fig 1. Description of the succession of the fish assemblages is presented in a recent overview of the Moroccan Post-Palaeozoic bony fish record [3], and here we focus on new material collected in the Ifezouane Fm.

Because most of the published fossils of vertebrates from the Ifezouane Formation have not been systematically excavated and have no precise geographical and stratigraphical associated data, it has been claimed that collecting biases strongly affect palaeoecological interpretations of this past ecosystem, especially when interpretations rest on the comparison of the relative proportions of taxa [4]. A recent analysis of samples of isolated vertebrate fossils systematically collected within a precise stratigraphical frame provides a better picture of the proportions of the vertebrate taxa found in the assemblage [5]. In particular, it has been shown that the proportion of aquatic and semi-aquatic organisms (including spinosaurid dinosaurs [6]) represents most of the identified vertebrate taxa (94.3%), and that piscine forms (chondrichthyans, actinopterygians, actinistia, dipnoans) are predominant (50% to 70% of the whole fossil vertebrate assemblage) in almost all fossiliferous levels of the Ifezouane Formation. This image, contrasting with the common picture of this assemblage known for its abundance of tetrapods, rests on the fact that isolated fish remains, mostly scales and isolated bones, are often neglected during campaigns of fossil collections. The fragmentary nature of most fish remains also makes difficult systematic determinations and provides systematic confusions. A recent description of vertebrate assemblages collected in coeval sedimentary levels located in the Guir basin, Western Algeria, from localities untouched by uncontrolled collections has shown similar large proportions of aquatic vertebrates than in the Moroccan localities [7].

It has been shown that the compound assemblage from the Ifezouane Formation is very similar to the assemblage from Bahariya in Egypt, originally studied by Stromer in 1925 [8] and in 1935 by Weiler [9] for the fish remains. Weiler [9] described with details the fish material, mostly represented by isolated and fragmentary remains, and the following year (1936) Stromer [10] discussed several Weiler’s determinations, and questioned some of them. Unfortunately, most of the fossil collection from Bahariya housed in Paläontologische Staatssammlung München was destroyed during World War II, which makes comparisons of the Moroccan material with the original Egyptian material impossible. Although based on material not available any more, Stromer and Weiler’s studies are taken into account in the present study because they have taxonomic consequences and shed light on the vertebrate assemblages of the Cenomanian of North Africa.

Here we describe new bony fish material collected during several field campaigns, we address their impacts on osteological reconstructions for several of the fish clades, and we discuss the systematic and taxonomic implications. We estimate the total body length for each taxon and reconstruct their trophic niches. Average body length versus average trophic level are plotted for the Kem Kem assemblage and compared with extant freshwater and marine bony fish assemblages.

## Material and Methods

Most of the new material described in this article has been collected during field campaigns conducted in the Kem Kem area in 2008, 2010, 2012 and 2013. The localities are plotted on a map and their stratigraphic locations are positioned in a simplified stratigraphical column (Fig 1). All the material collected in situ is from the Ifezouane Formation, which corresponds to the lower part, or unit 1, of the informally called Kem Kem beds [11]. The Ifezouane Fm. is mostly composed of essentially consolidated sandstones with cross-bedding structures. The fossils are often, but not always, concentrated in the bottom of channels within micro-conglomerates. Erosion weathers the outcrops of the Ifezouane Fm. and washes out the fossils, which then lay on the ground. Most of the specimens were collected by picking up these fossils. The largest specimens, in particular braincases of *Concavotectum* described below, have been excavated in the site of Tizi Momrad during the 2008 field campaign. For authorisation and support of our fieldwork we thank the Ministère de l’Energie, des Mines, de l’Eau et de l’Environnement of Morocco and the Faculté des Sciences of the University Moulay Ismail of Meknès, Morocco. Most of the collected specimens are housed at the University Moulay Ismail of Meknès, Morocco (UMI), four specimens at the Société d’Etude des Sciences Naturelles d’Elbeuf, France (SESNE) and four specimens described in the paper are curated at the Musée des dinosaures d’Espéraza, France (MDE). All the specimens are openly accessible to any interested researcher at these locations. A list of material with repository information is available (S1 Text).

Predator-prey relationships of extinct fish communities are better reconstructed on the basis of fossil gut contents indicating direct evidence of predation. For the Mesozoic, this approach was made possible with the very rich and well preserved Santana and Solnhofen assemblages ([12] and Viohl, 1990 cited in [12], respectively). However, trophic behaviours may also be reconstructed on the basis of indirect evidence (Boucot, 1990 cited in [12]), such as functional interpretation of morphology (Boucot’s category 2B), which is the main approach used here. Based on these data, we reconstructed the trophic niches of each taxon from the Kem Kem assemblage. Then, each taxon is assigned to a trophic level as defined in Fishbase (Fishbase.org [13]). Trophic levels are represented by a scale ranging from 1, the primary producers or plants, to 5, the top predators. Because carnivorous organisms may feed partly on plant and on a variety of prey items, which may belong to different trophic levels, Pauly and Palomares [14] defined the trophic level of a consumer species *i* as:

$${TL}_{i}\;=\;1+\;\sum_{j}\left({TL}_{j}\times {DC}_{ij}\right)$$

Where *TL*_*j*_ is the fractional trophic level of the prey _j_, and *DC*_*ij*_ represents the fraction of *j* in the diet of *i*. This value cannot be directly calculated for extinct species. Accordingly, we estimated the TL value of taxa from the Kem Kem assemblage by comparison with TL provided by Fishbase [13] for taxonomically and/or ecologically close taxa.

The trophic network of the Kem Kem assemblage is then compared with reconstructed networks for the Santana Formation assemblage [9], and for the Goulmima assemblage [15], which are both relatively close in space and age with the Kem Kem assemblage.

We also estimated the total length of the Kem Kem taxa represented by incomplete specimens by comparisons and extrapolations with phylogenetically close species known by well-preserved specimens. Fishbase provides lists of bony fish species for the extant assemblages, which are compared with the Kem Kem assemblages, as well as the average total length for the each taxon present in these assemblages. Comparison between extinct and extant assemblages (six freshwater faunas: Amazon basin, Congo basin, Ganges, Mississippi complex, Okawango, Paraguay basin, and two marine assemblages: Atlantic Ocean, Mediterranean Sea) is done by plotting the average trophic level of each assemblage against the mean total length of the species composing each assemblage. We are aware that the marine assemblages used here for comparison encompass a broad array of different ecosystems, but we regarded them as suitable approximations for this very general comparison.

## Results

### Systematic Palaeontology

Sarcopterygii Romer, 1955

Dipnoi Müller, 1845

#### Remark

Three different lungfishes at least are present in the Kem Kem assemblage. Their supra generic assignments are not definitively resolved ([16], [17], [18]), but this issue is not addressed here.

Ceratodontidae Gill, 1872

*Ceratodus humei* Priem, 1914

#### Description of new material

**Fig 2:**
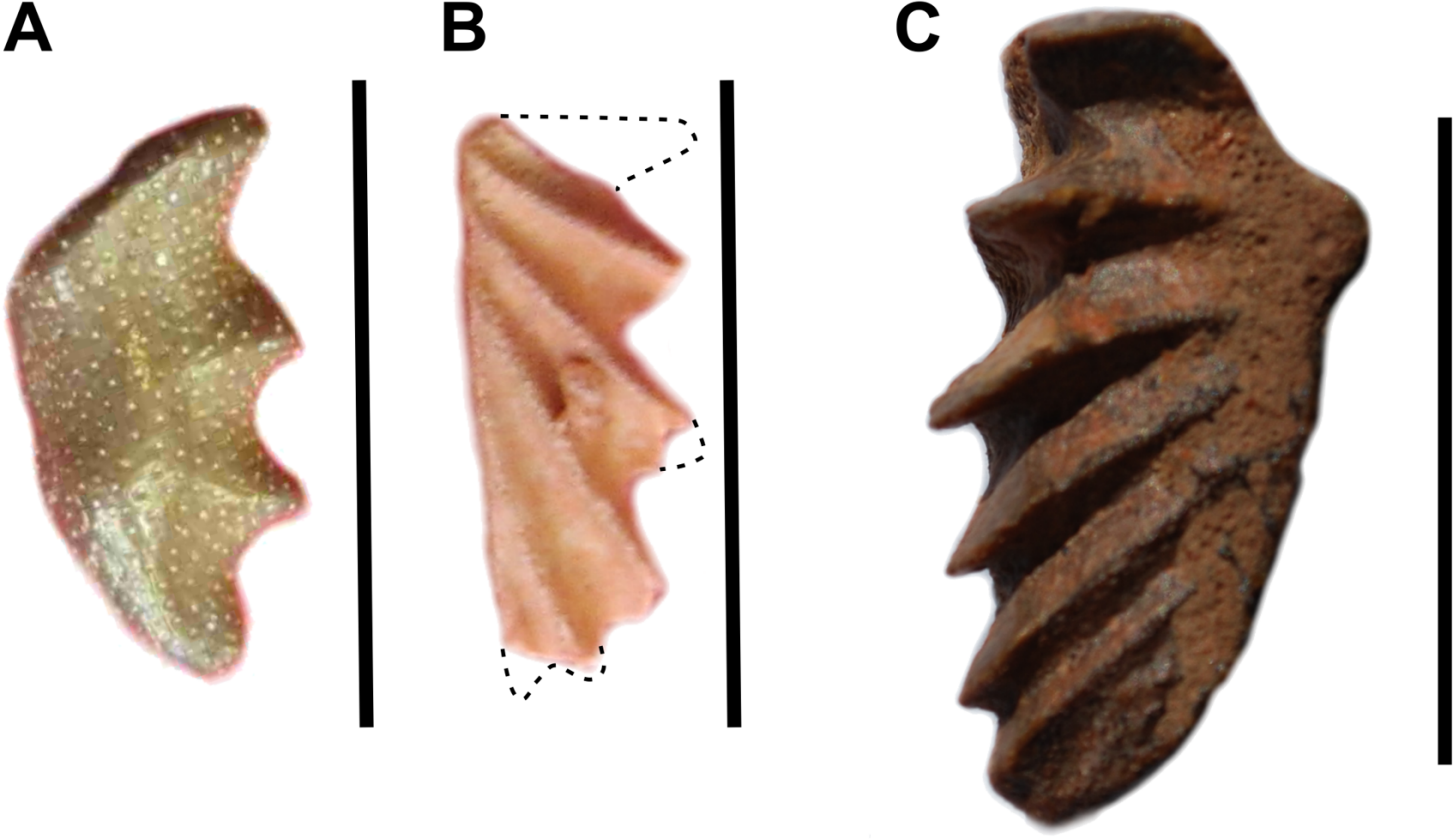
Dipnoi, tooth plates. A, *Ceratodus humei* (UMI-4); B, *Arganodus tiguidiensis* (UMI-2). Dotted lines show the reconstructed silhouette; C, *Neoceratodus africanus* (UMI-3). Scale bars: A and B, 10 mm; C, 20 mm.

A small tooth plate of *Ceratodus humei* (Fig 2A, UMI-4) was found in Douira. It is characterized by a masticatory surface almost flat with no crests, four denticulations on the labial margin, a straight mesial margin and a curved lingual margin. These features are diagnostic for *C*. *humei* [16].

Arganodontidae Martin, 1982

*Arganodus tiguidiensis* (Tabaste, 1963)

#### Remark

This corresponds to the first mention of *Arganodus tiguidiensis* in the Ifezouane Formation. This species was originally described from Algeria [16], then recorded in Niger [19] and eventually in the Alcântara Formation in Brazil ([20], [21]).

#### Description of new material

A very small (the preserved portion is 8 x 4 mm) tooth plate (Fig 2B, UMI-2) anteriorly and laterally broken was found in Chaaft (level 1). Four straight well developed crests oriented posteriorly are preserved. The medial extremity of a first crest, oriented almost at right angle with the internal margin of the plate, as well as the departure of a posterior crest, indicate that 6 crests were originally present. The right angle shape of the tooth plate (before damage), the length being likely twice longer than the width, the radiating straight ridges oriented posteriorly (except the first one) are specific characters for *Arganodus tiguidiensis* [16]. In this species, the lower tooth plates have generally 7 crests and the upper tooth plates have generally 8 crests, but one tooth plate described by Tabaste has 6 crests, as in our specimen.

Neoceratodontidae Schultz, 1948

*Neoceratodus* De Castelnau, 1876

*Neoceratodus africanus* (Haug, 1905)

#### Description of new material

Tooth plates of *N*. *africanus* of various size have been found in Chaaft (level 1; Fig 2C, UMI-3), in ANG W (level C, UMI-20) and in Khetitila (UMI-7, still attached to the pterygoid). They are referred to *N*. *africanus* because they are longer than wide, they have from 5 to 7 crests, the crests, except the first two, are oriented posteriorly since their origin and the lingual margin is curved [16]. An isolated pterygoid, referable with caution to *N*. *africanus* because of the elongated sutural surface for the tooth plate, has been found in ANG, level B (UMI-5). One angular (or ‘labial bone’ according to Cavin et al.’s nomenclature [22]) was found near Chaaft (UMI-8) and another one, with a fragmentary tooth plate still attached, was found in ANG W (C) (UMI-22).

Actinistia Cope, 1871

Mawsoniidae Schultze, 1993

*Mawsonia lavocati* Tabaste, 1963

#### Remark

*Mawsonia* is a genus of usually large coelacanths originally described from South America [23], then in several North African Early Cretaceous and Cenomanian localities. In the Ifezouane Fm., *Mawsonia* is known with *M*. *lavocati* [16],[24,25,26] and by remains reminiscent of the South-American sister-genus of *Mawsonia*, *Axelrodichthys* [27]. In 1935, Weiler [9] described *Stromerichthys aethiopicus* on the basis of material found in the Cenomanian of Bahariya in Egypt. The referred material is “a series of unidentified head bones found associated with jaw bones and bones of the opercular series, which most likely are from a single individual, because they fit each other, they are of comparable size and several are preserved in pairs” (translated from German). Weiler’s Fig 2 illustrates opercular bones, which apparently fit together and are rather similar to dermal bones of *Mawsonia*. Weiler distinguished the opercle of *Stromerichthys* by being proportionally thinner than the opercle of *Mawsonia* and by the different shape of its anterodorsal edge. Stromer [10] questioned some of Weiler’s identification of *Mawsonia*, but he accepted the distinction between *Mawsonia* and *Stromerichthys* based on the opercle (his Fig 1: 39). We question these differences here and suspect that the opercle of *Stromerichthys* illustrated in Weiler’s Fig 2 belongs to a mawsoniid (keeping in mind that probably several taxa are present), and reject the identification of the subopercle and preopercle illustrated in the same figure. The ‘interopercle’ shows a different ornamentation, honeycomb-like, and we question here both its anatomical and systematic identifications. As shown below, it is likely that ‘*Stromerichthys aethiopicus*’ is a taxon erected on a mixture of ossifications belonging to different taxa.

Gen. et sp. indet.

#### Description of new material

**Fig 3:**
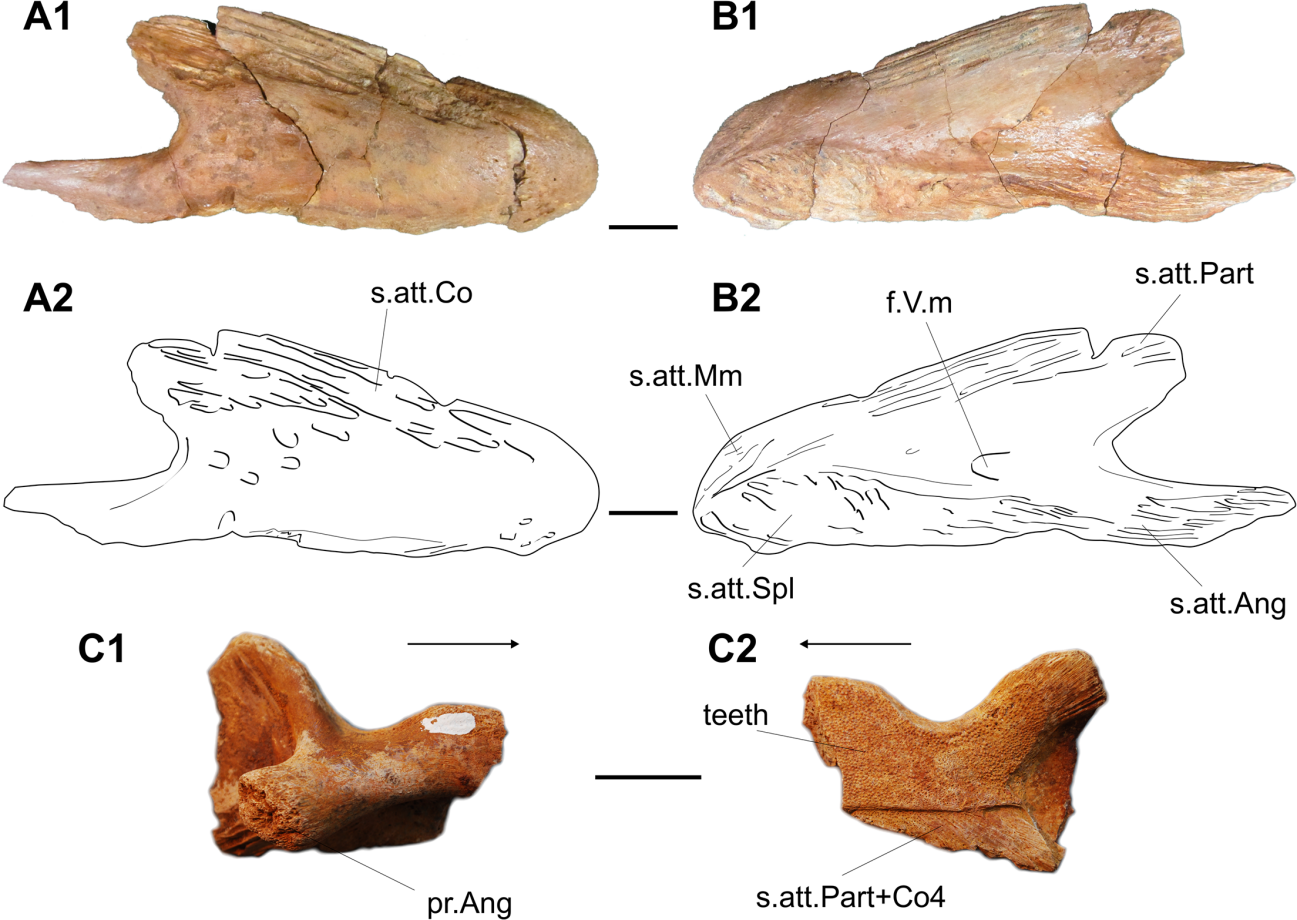
Mawsoniidae. A-B, Mawsoniidae gen. et sp. indet. Right dentary (UMI-1). 1, photographs and 2, line drawings in lateral (A) and medial (B) views. C, cf. *Axelrodichthys*. Principal coronoid (UMI-30) in lateral (1) and medial (2) views. Arrows point anteriorly. f.V.m, foramen for the mandibular branch of the V^th^ nerve; pr.Ang, process sutured with the angular; s.att.Ang, surface of attachment for the angular; s.att.Co, surface of attachment for the coronoid; s.att.Mm, surface of attachment for the mentomeckelien; s.att.Part, surface of attachment for the prearticular; s.att.Part+Co4, surface of attachment for the prearticular and fourth coronoid; s.att.Spl, surface of attachment for the splenial. Scale bar: 20 mm.

An almost complete isolated ossification from ANG, 162 mm of maximum length, is regarded as a large right dentary (Fig 3A and 3B, UMI-1). The main body of the bone is ovoid in lateral view, with no ornamentation except a few oval pits in the posterior half. A well-developed hook-shaped process extends posteroventrally from the main body of the bone, which fitted in a ridge of the angular. Along the ventral border of the internal face run strong ridges that mark the surface of attachment for the splenial anteriorly and angular posteriorly. Along the dorsal margin of the bone are also ridges, possibly for the attachment of the 4^th^ coronoid on the lateral face and, on the internal face, for the prearticular posteriorly and for the mentomeckelian anteriorly. Almost in the middle of the medial face of the dentary opens a large pore for the mandibular branch of the V^th^ nerve.

This dentary shares with dentaries of both *Mawsonia* and *Axelrodichthys* the well-developed hook-shaped process, but it differs by the lack of a depression for the pseudomaxillary fold and bears no well-developed lateral swelling anteriorly.

UMI-16, from Chaaft (level 1) is a triangular flat bone with the typical mawsoniid ornamentation regarded as a possible preopercle or subopercle.

cf. *Axelrodichthys*

UMI-30 is a right principal coronoid from an unknown locality (Fig 3C). It shows characters of mawsoniids [28,29] and in particular those listed by Forey [30]: a saddle shape, the posterior end rises higher than the anterior end, there is a clear ventral overlap surface along the ventral edge for the prearticular and the coronoid 4. Laterally a process extends posteriorly to suture with the angular, which is a synapomorphy of *Axelrodichthys* and *Mawsonia* [30]. The angular process is stout as in *Axelrodichthys*, while it is smaller in *Mawsonia*. This principal coronoid can be referred with caution to *Axelrodichthys*, thus confirming the occurrence of this genus in North Africa [27].

Actinopterygii Cope, 1887

Cladistia Cope, 1871

Polypteriformes Bleeker, 1859

Polypteridae Bonaparte, 1835

cf. *Bawitius* Grandstaff, Smith, Lamanna, Lacovara & Said Abdel-Ghani, 2012

#### Remark

In 1935, Weiler [9] referred to *Stromerichthys aethiopicus* several remains, among them two maxillae. The ossifications are characterized by a gently convex oral margin, a widened posterior lamina, an internally sutural surface extending backwards, possibly for the pterygoid, a sutural contact dorsally for a supramaxilla (sic), and six conical teeth, slightly inwardly curved and topped by cup of enameloid. This description fits well with the description of the maxilla of *Polypterus* by Allis [31]: the bone bears a single row of stout sharp teeth, the internal lamina sutures with the dermopalatine and the anterior extremity of the ectopterygoid, the dorsal margin of the bone is in contact with the postorbital and one suborbital and the posterior extremity of the ossification widens. In the fossil material, no sensory canal is mentioned through the bone and the number of teeth, 6, is less than in *Polypterus* (circa 15).

**Fig 4:**
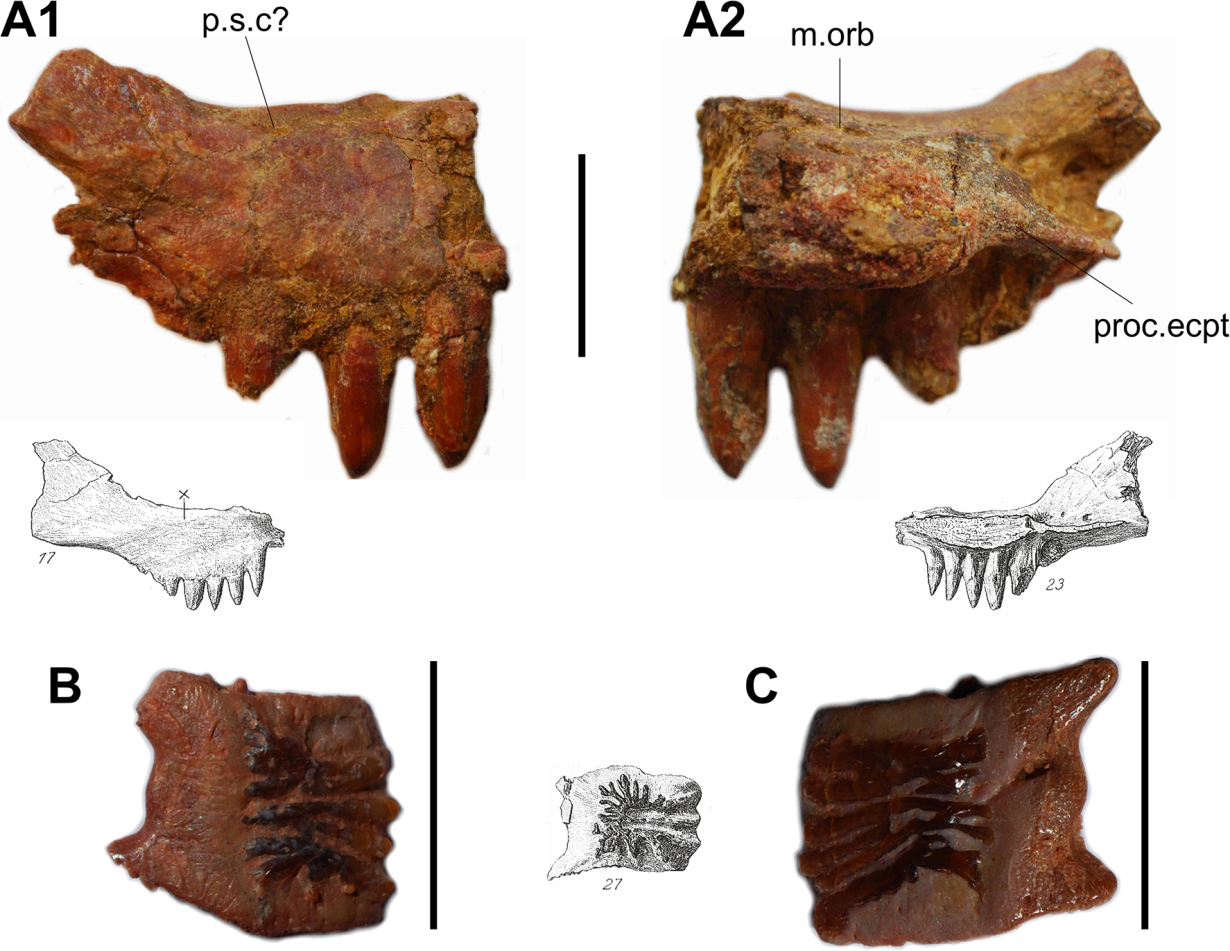
cf. Bawitius. A, Posterior part of a maxilla (SESNE 1-03-2008). Photographs in lateral (1) and medial (2) views. m.orb, margin of the orbit; proc.ecpt, processus ectopterygoideus; p.s.c?, pore of the sensory canal?. B and C, scales of cf. *Bawitius* (UMI-12). Scale bars: 20 mm. Grey drawings are corresponding elements in Weiler [9], plate III (not to scale).

**Fig 5:**
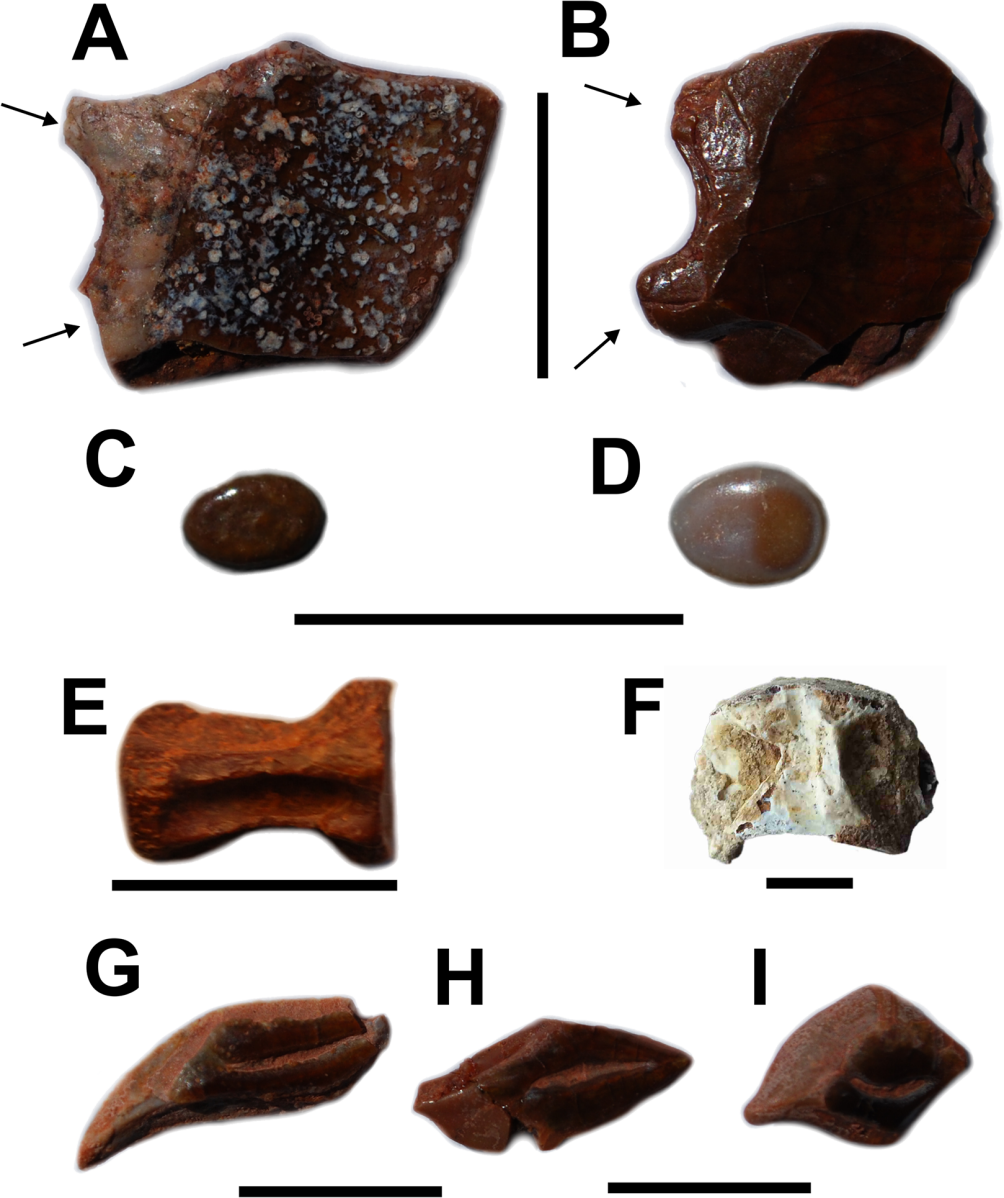
Isolated ganoid scales, teeth and vertebral centra. A-B: cf.? *Lepidotes pankowskii*, isolated scales. Arrows indicate the anterior processes (UMI-13). C-D:? *Lepidotes pankowskii*, isolated teeth (SESNE 1-61-2008). E-F: indeterminate Lepisosteoidea centra, E: caudal centrum (UMI-9), F: abdominal centrum (MDE-F-60). G-I:? *Dentilepisosteus kemkemensis* (UMI-11), isolated scales. Scale bars: 10 mm.

In 1925, Stromer [8] referred isolated scales and bones from Bahariya to a polypterid, scales that Weiler [9] regarded later as belonging to *Lepidotus* aff. *souzai*. Soon later, Stromer [10] maintained his initial identification, in particular on the basis of new histological observations. Schaal [32] described from the same locality the species *Polypterus*? *bartheli* on the basis of an isolated ectopterygoid, that he included without doubt to the family Polypteridae (but he referred it with caution to the genus *Polypterus*). Schaal also described teeth of a polypterid that he attributed to a genus and species indet. The gross morphology and the histology of new scales from Bahariya were described by Smith et al. [33] and by Grandstaff et al. [34]. The latter authors referred *P*.? *bartheli* to a new genus, *Bawitius*. In Morocco, Tabaste ([16] pl. XI, Figs 1–2, 4–5) described some isolated scales from Gara Tabroumit (a locality situated ca 25 km SSW of Gara Sbâa) as *Lepidotes* sp., but these specimens may actually belong to *Bawitius bartheli*. Isolated scales from the Albian of the Democratic Republic of Congo may be referred to *Bawitius* sp. as well [35]. Benyoucef et al. have recently recorded in the Guir basin, Western Algeria, relatively small scales with a similar morphology to Bawitius [7].

Because the original material of *Stromerichthys* is now destroyed, *Bawitius* cannot be regarded as a junior synonym of *Stromerichthys*.

#### Description of new material

A fragment of an ossification, with two complete teeth and the base of two others, has been discovered in 2008 in the locality of Tizi Moumrad (SESNE 1-03-2008) (Fig 4). Isolated teeth of similar size and shape have been found in various other localities. These teeth can be referred to the teeth of type 1 of *Polypterus* according to Clemen et al’s classification [36], i.e. massive, broad-based teeth with a distinct enameloid cap that occupied approximately 25% of the tooth length (or a little less in SESNE 1-03-2008). The base of the posterior-most tooth, smaller than the anterior ones, is located at the level where the bone is narrowing, which corresponds to the level of articulation of the processus ectopterygoideus [36,37]. In polypterids, the maxilla is regarded as the fusion of a circumorbital ossification with the maxilla, as indicated by the path of a sensory canal [31]. As in *Polypterus*, the dorsal margin of the fragment described here is concave and probably formed the margin of the orbit. The beginning of the thinner posterior portion projecting posterodorsally in *Polypterus* in still preserved on SESNE 1-03-2008. Several pores, whose functions are unclear, open on the dorsal and posterior part of the preserved fragment. One foramen, situated on the dorsal margin at the level of the posterior curve, may correspond to the posterior opening of the sensory canal, present in a rather similar location in *Polypterus* [31].

The large size of the maxillary described here fits well with the gigantic size of the ectopterygoid of *Bawitius*, and we suspect that they may belong to a closely related or to the same taxon.

Ganoid scales (Fig 4B and 4C) with an incomplete layer of ganoin forming reticulated ridges are commonly found (UMI-12, -21, -28). The posterior margin forms right angles with the dorsal and ventral margins, making the general outline squarish rather than rhomboidal as it is usually the case in most holosteans and extant bichirs. In some scales (UMI-21), an articular process extends anterodorsally from the anterodorsal corner of the scale and in other scales this process is oriented more anteriorly, and a second anterior process may extend from the anteroventral edge of the scale (Fig 4B and 4C, UMI-12).

The general morphology of these scales are similar to those described from Bahariya [10,32,33,34], and we follow these authors in referring them *Bawitius*. The histology of these scales is currently under study.

*Bartschichthys* sp.

*Sudania* sp.

#### Remark

*Bartschichthys* and *Sudania* are two genera of polypterids identified by Dutheil [2] on the basis of isolated fin spines. These taxa were originally described on the basis of the morphology of isolated fin spines from the Coniacian-Santonian of Niger and from the Cenomanian of Sudan, respectively [38],[39]. More complete material is pending in order to test if these fin spines correspond to genera distinct from *Bawitius*.

Neopterygii Regan, 1923

Holostei Müller, 1844 (*sensu* Grande 2010)

Ginglymodi Cope, 1872 (*sensu* Grande 2010)

Lepisosteiformes Hay, 1929 (*sensu* López-Arbarello, 2012)

Family indet.

?*Lepidotes pankowskii* Forey, López-Arbarello & MacLeod, 2011

#### Remark

This species was defined on two isolated partial skulls from an unknown locality in the Ifezouane Fm. [40]. Following the new stem-based definitions of ginglymodian clades by López-Arbarello [41],? *L*. *pankowskii* can be included in the Lepisosteiformes because: the anterior-most supraorbital bone is trapezoidal, longest ventrally and contacts more than one infraorbital bone; the vomers are co-ossified; there are three or more pairs of extrascapular bones; the circumborbital ring is closed and supraorbital bones are large. The single character that this species does not share with Lepisosteiformes is a ventral border of the infraorbital series, which do not flex abruptly dorsally at the anterior margin of the orbit. We suppose that this character state is a reversion.? *L*. *pankowskii* does not possess several characters defining the genus *Lepidotes* according to López-Arbarello’s revised diagnosis [41], such as: the first anterior infraorbital bone deeper than more posterior anterior infraorbitals; the presence of a single pair of extrascapular bones and the suborbital bones pattern is very different. Consequently, we regard? *L*. *pankowskii* as a lepisosteiform not belonging to *Lepidotes*, but further phylogenetic studies are necessary to assess its relationships.

#### Description of new material

Isolated scales with a smooth ganoin surface and with two processes on the anterior margin, typical in most non-gar Lepisosteiformes [41,42] are regularly found in several localities (Fig 5A and 5B, UMI-13). They are referred with caution to? *L*. *pankowskii*. Tiny ovoid crushing teeth (Fig 5C and 5D, SESNE 1-61-2008) are regularly found. They are referred to? *L*. *pankowskii* because they are similar to the vomerine teeth of this species described by Forey et al. [40].

Lepisosteoidei López-Arbarello, 2012

Lepisosteoidea Bonaparte, 1835 (sensu López-Arbarello, 2012)

Lepisosteoidea indet.

#### Description of new material

Isolated opisthocoelous piscine vertebral centra are not uncommon in the Ifezouane Fm. and may be attributed without doubt to lepisosteoids, i.e. gars (lepisosteids) or obaichthyids (Fig 5E and 5F, UMI-9 and MDE F-60).

Obaichthyidae Grande, 2010

*Obaichthys* Wenz & Brito, 1992

*Obaichthys africanus* Grande, 2010

#### Remark

*Obaichthys africanus* is a species described by Grande [43] on the basis of isolated remains (scales and one basioccipital) from the Kem Kem beds. Grande referred this material to the genus *Obaichthys*, whose type species *O*. *decoratus* from the Santana Formation in Brazil is known by well-preserved articulated specimens, on the basis of the basioccipital morphology. The basioccipital of both species bears elongated lateral processes, which is a uniquely derived feature for ginglymodians. A character of the scales present in both species, presence of posterior marginal spines, is diagnosable of the family Obaichthyidae (containing *Obaichthys* and *Dentilepisosteus*). The morphology of the scales of *O*. *africanus* from the Kem Kem assemblage corresponds to those referred to *Stromerichthys aethiopicus* by Weiler [9] from the Cenomanian of Bahariya in Egypt. This taxon rests on a mix of fish remains referable to a polypterid and possibly a mawsoniid coelacanth (see above), and consequently the name of this genus cannot be kept for these scales.

Scales of *Obaichthys* have been recorded for a long time under the name *Stromerichthys* in the Cenomanian of Morocco [2,16], as well as in other mid-Cretaceous localities, such as the Albian of the Democratic Republic of the Congo [35], the Cenomanian of southwest France [44], Portugal [45,46,47] and Spain [48,49]. Casier also described from the Albian beds in the Democratic Republic of Congo a fragmentary bone, which he referred with caution to a coelacanth [35]. The ossification is covered with well-developed posteriorly inclined denticles, whose tip is not in contact with the supporting bone. Such ‘teeth’ borne by the dermal skull bones are reminiscent of *O*. *decoratus* from the Albian of the Santana Fm., but differ from the situation in *O*. *africanus*, in which patches of ganoin always adhere to the surface (see below). This similarity between the Congolese and the Brazilian forms are in accordance with their supposedly similar age (Albian), in contrast with the slightly younger age of the Kem Kem assemblage. Recently, scales referred to *O*. *africanus* were reported from the Guir basin [7].

#### Description of new material

**Fig 6:**
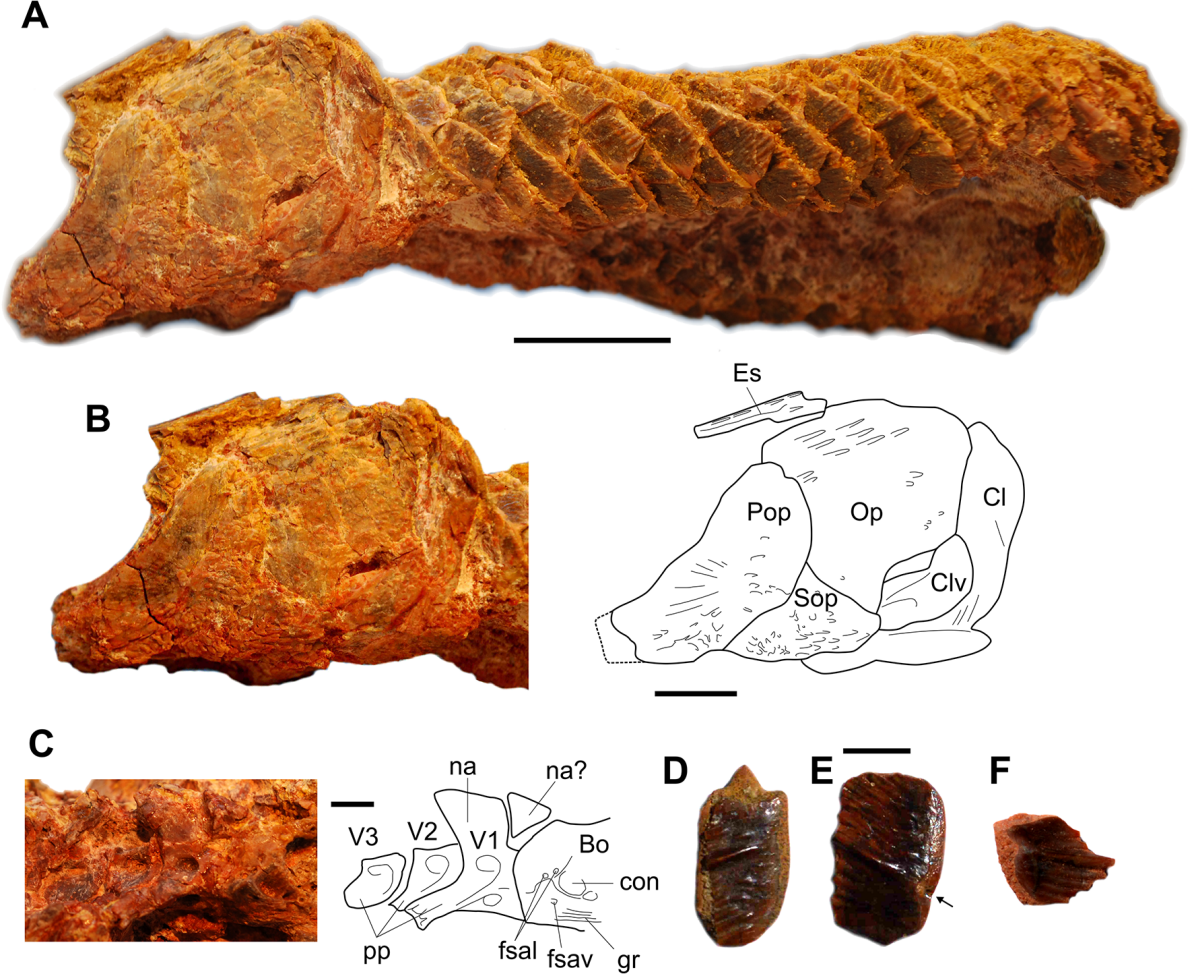
*Obaichthys africanus*. A – C, partly articulated specimen (UMI-31). A, left lateral view of the complete specimen. B, photograph and drawing of the head in left lateral view. C, photograph and drawing of the posterior part of the braincase and three first vertebrae in left lateral view. D-F, isolated scales [UMI-17 (D, E) and UMI-14 (F)]. Bo, basioccipital; Cl, cleithrum; Clv, clavicle; con, concavity; Es, extrascapular; fsal, lateral foramen; fsav, ventral foramen; gr, groove; na, neural arch; Op, opercle; Pop, preopercle; pp, parapophysis; Sop, subopercle; V, vertebra (numbered). Scale bars: A, 20 mm; B, 10 mm; C-F, 5 mm.

UMI-31 (Fig 6A and 6B) is an articulated specimen preserving about half of the anterior part of the body, without fins, and the posterior part of the head, which comes from an indeterminate locality of the Ifezoune Fm. The scales are diagnosable for *O*. *africanus*, i.e. ‘the ornamentation on the scales consists of elongate, flattened ganoid lines and grooves, versus a fine pebbly surface of small rounded tubercles in *O*. *decoratus*’ [43]. The left side of the head shows the opercular series and part of the pectoral girdle, and the right side shows some poorly preserved ossifications of the braincase and the first vertebrae. The left lateral-most extrascapular is partly preserved and bears elongated patches of ganoin. Similar patches are present on the posteroventral corner of the preopercle, on the dorsal part of the opercle and on the subopercle, and more irregular shaped patches are scattered on the opercle and preopercle. Grande [43] mentioned in *O*. *decoratus* and *Dentilepisosteus* ‘large conical teeth on the external surface on many of the dermal skull bones’, while here the ganoid always adhere to the surface of the bone and does not form real teeth, with a detached apex. We cannot decide if this difference is due to the fact that only the posterior-most part of the skull is preserved in our specimen (the anterior part may have borne such teeth), or if this a taxonomic difference.

The limits of bones in the opercular series are difficult to detect. As in other obaichthyids, the preopercle has a vertical limb exposed to the surface (in lepisosteids, the vertical branch is generally hidden by the suborbitals.) The dorsal extremity of the bone is broad and rounded, with a shallow notch in the posterior part, while in *O*. *decoratus* the upper part of vertical limb is narrower with parallel margins. The anterior margin of the bone curves regularly, with no marked angle between the vertical and the horizontal limbs, and the posterior margin is almost parallel to the anterior margin. In *O*. *decoratus*, the anterior margin of the bone has a similar curvature, but the posterior margin formed a right angle. The crescent shape of the preopercle of *O*. *africanus* should be regarded as diagnosable for this species. The opercle is a large rounded bone, almost as long as deep. The subopercle is preserved as a triangular bone wedged between the anteroventral corner of the opercle and the posteroventral margin of the preopercle. It does not extend along the ventral margin of the opercle, but this may be due to damages in this area. No anterodorsal process of the subopercle, very pronounced in *O*. *decoratus*, is visible on the specimen. But it is unclear if this feature is due to a shift of the bone or if it corresponds to a real difference between both species. A broken ventral portion of the opercle allows observing a proportionally large ridged ossification resting on the cleithrum and extending under the subopercle anteriorly. We follow Grande, who considers an ossification located in a similar position in *O*. *decoratus* as a probable clavicle ([43]: 680 and 686).

Preparation of the right side of UMI-31 revealed the posterior-most part of the braincase and the first three vertebrae. The basioccipital shows a deep concavity on its lateral face, but not the well-developed lateral process typical of *Obaichthys*, also observed on the type material of *O*. *africanus* [43]. We suspect that, because only the basal part of the basioccipital is preserved, the process should have been located slightly more dorsally and is now destroyed. Two foramina open above the deep concavity and one in ventral position for the spinal artery. The anterior ventral portion of the basioccipital bears deep grooves for the lap suture (sensu [43]) with the parasphenoid. The first three vertebrae have deep pits located dorsally and ventrally to the parapophyses. The parapophyses developed laterally into prominent broad blades. So far, this character was observed in *O*. *decoratus* only ([43]: 682) and we can confirm that it is diagnostic for the genus *Obaichthys*. The neural spine of the first vertebra only is preserved. As in *O*. *decoratus*, this neural spine is proportionally broad within the sagittal plane. A small triangular ossification is wedged between the basioccipital and the neural arch of the first autogenous vertebra. It possibly corresponds to the neural arch of a centrum fused with the basioccipital.

Typical scales of *O*. *africanus* (formerly *Stromerichthys*) are regularly found in several localities (Fig 6D, 6E and 6F). The histology of these scales is currently under study.

*Dentilepisosteus* Grande, 2010

?*Dentilepisosteus kemkemensis* Grande, 2010

#### Remark

This species was defined by Grande [43] on the basis of material from the Ifezouane Fm., which consists of isolated scales and a piece of a trunk region with scales and four vertebrae. An isolated scale from Gara Tabroumit (a locality from the Kem Kem region), described by Tabaste ([16]: pl. XI, Fig 3) as *Lepidotes* sp., can be referred to? *Dentilepisosteus kemkemensis*. In addition, similar scales were described from the Cenomanian of Portugal under the names ‘*Paralepidosteus cacemensis*’ and ‘*Lepidotes minimus*’ (*nomina dubia*) ([46]: pl. 2, Figs 43–44). Jonet [46] erected ‘*Paralepidosteus cacemensis*’without having provided any diagnosison the basis of various isolated elements not proved to belong to a single species and consisting of two vertebrae (including the type specimen), six scales, and one jaw fragment. Scales referred to? *D*. *kemkemensis* were reported from the Guir basin [7].

#### Description of new material

Isolated scales found in Chaaft (level 1) (Fig 5G, 5H and 5I, UMI-11) display elongated patches of ganoin separated by a groove, in which the bony basal plate is visible. This morphology is very similar to the scale of? *Dentilepisosteus kemkemensis* described by Grande [43]. Although the gar origin of this species is demonstrated by the presence of associated opisthocoelous vertebrae in the type specimen, the morphology of these scales are rather different from the type species, *D*. *laevis* from the Santana Formation in Brazil.

Lepisosteidae Bonaparte, 1835 (sensu López-Arbarello, 2012)

*Oniichthys* Cavin & Brito, 2001

*Oniichthys falipoui* Cavin & Brito, 2001

#### Remark

*Oniichthys falipoui* is a fossil gar described on the basis of two specimens from the Ifezouane Formation [50]. The genus was originally regarded as distinct of both Recent gars genera, *Lepisosteus* and *Atractosteus*, because of the presence of a toothed maxilla, of few postorbital plates and of the lack of plicidentine on the lachrimomaxillar (or ‘infraorbital’ according to Cavin & Brito’s nomenclature) teeth. Grande [43] re-described this species and considered that the toothed maxilla does not differ from the posterior maxillary projection of the posterior-most lachrimomaxilla in living gars and, after preparation of ‘cross-sections’, he showed that plicidentine is actually present on the lachrimomaxillar teeth. The small bony elements in the suborbital area were regarded by Cavin & Brito [50] as fragments of a few, possibly two, broken ossifications while Grande [43] regarded them as independent ossifications, probably 10 in number. Although this amount is lower than in most Recent gar species, Grande considered that this character, together with the other mentioned above, are evidence that this species should be included in the genus *Atractosteus*.

#### Description of new material

**Fig 7:**
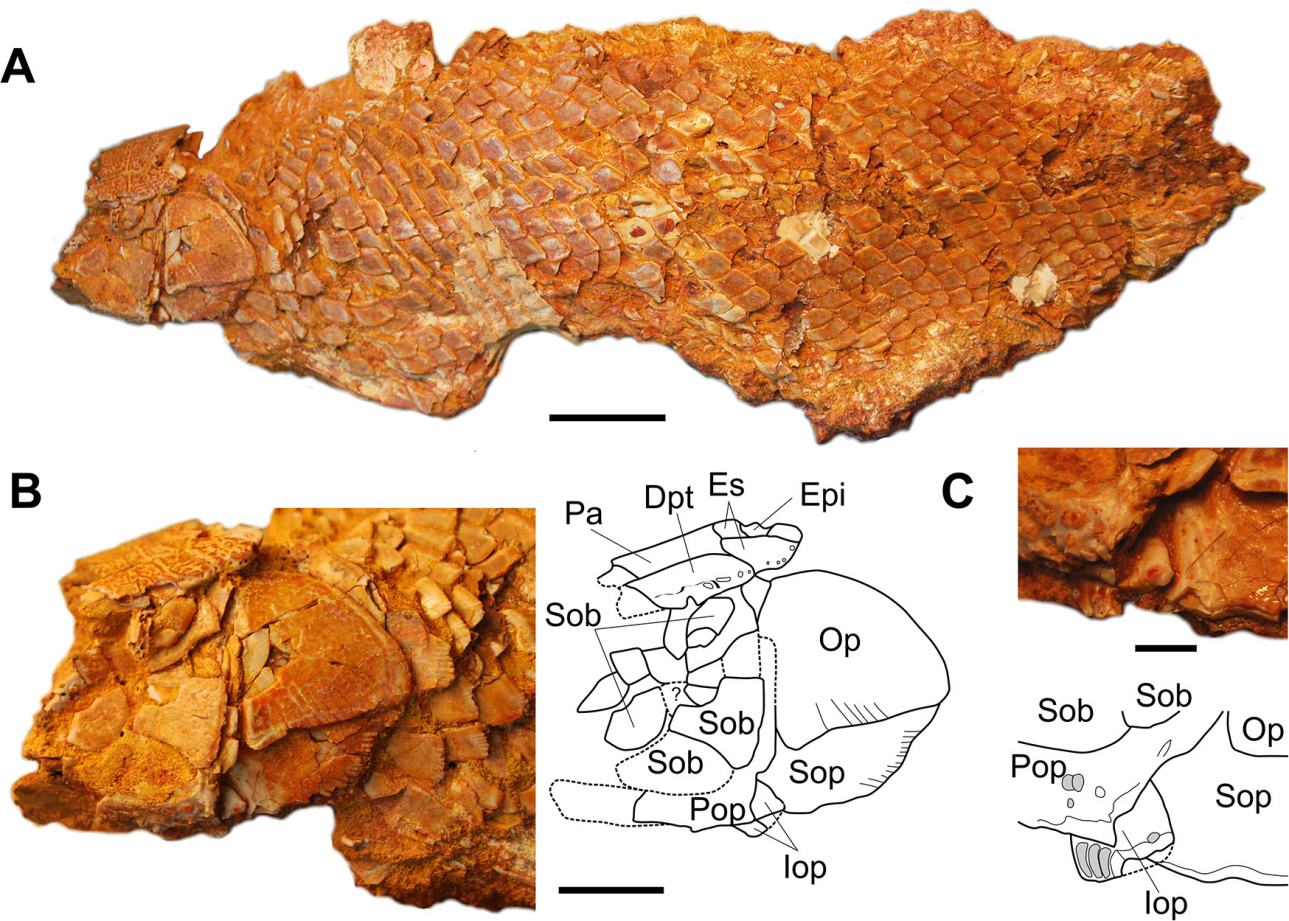
*Oniichthys falipoui* (MDE F-13). A, left lateral view of a partly articulated specimen. B, photograph and drawing of the head in left lateral view. C, photograph and drawing of the ventral area of the opercular series in left lateral view. Dpt, dermopterotic; Epi, epioccipital; Es, extrascapular; Iop, interopercle; Op, opercle; Pa, parietal; Pop, preopercle; Sob, suborbital; Sop, subopercle. Scale bars: A, 20 mm; B, 10 mm; C, 3 mm.

MDE F-13 (Fig 7) is an articulated specimen with the tail, the fin and the anterior two-thirds of the skull missing from an unknown locality in the Ifezouane Fm. The scales are smooth, the medial extrascapulars are larger than the lateral extrascapulars and the subopercle has a fringed ventral margin, three characters diagnostic for *Oniichthys falipoui*. The left suborbital region is preserved but damaged. Two large suborbitals (one preserved as an imprint) are visible in the posteroventral region, bordering the inner angle of the preopercle, in a similar arrangement as in the paratype. The number and arrangement of suborbitals is uncertain, but there are probably 10 or more plates (one possibly being a fragment of a postorbital, or ‘posterior infraorbital’ according to Cavin & Brito’s nomenclature). The left opercular series is well preserved and consists of a large opercle, a fringed subopercle and a small interopercle. The latter bone is located in the anteroventral corner of the opercle and extends anteriorly under the horizontal branch of the preopercle. It bears some small patches of ganoine. Although broken, this bone, which was not observed on the holotype and paratype specimens, cannot be mixed up with another ossification. Fragments of the braincase are visible on the right side and we recognise the posterior extremity of the parasphenoid extending to the occipital condyle, the basioccipital, the exoccipital and the epioccipital, but details are difficult to describe without more preparation.

The new specimen confirms that the number of suborbital in this species is superior to two, as suggested by Grande [43]. However, the occurrence of an interopercle is an indication that this species cannot be referred to one of the living (*Lepisosteus*, *Atractosteus*) or extinct (*Masillosteus*, *Cuneatus*) genera of lepisosteids, a family characterized by the absence of interopercle [43]. The obaichthyids have an interopercle but *O*. *falipoui* shows no autapomorphy of this clade. Pending new discoveries and further phylogenetic analysis, *O*. *falipoui* is regarded as a probable stem lepisosteid according to López-Arbarello’s stem-based definition of this family [41].

Halecomorphi Cope, 1872

Amiiformes Hay, 1929

Amiidae Bonaparte, 1831

Vidalamiinae Grande & Bemis, 1998

Calamopleurini Grande & Bemis, 1998

*Calamopleurus* Agassiz, 1841

*Calamopleurus africanus* Forey & Grande, 1998

#### Remark

This species, regarded as the twin species of *Calamopleurus cylindricus* from the Santana Formation, Brazil, was described on the basis of a crushed head and some isolated remains from an indeterminate locality of the Ifezouane Fm. from the Taouz area [51].

#### Description of new material

**Fig 8:**
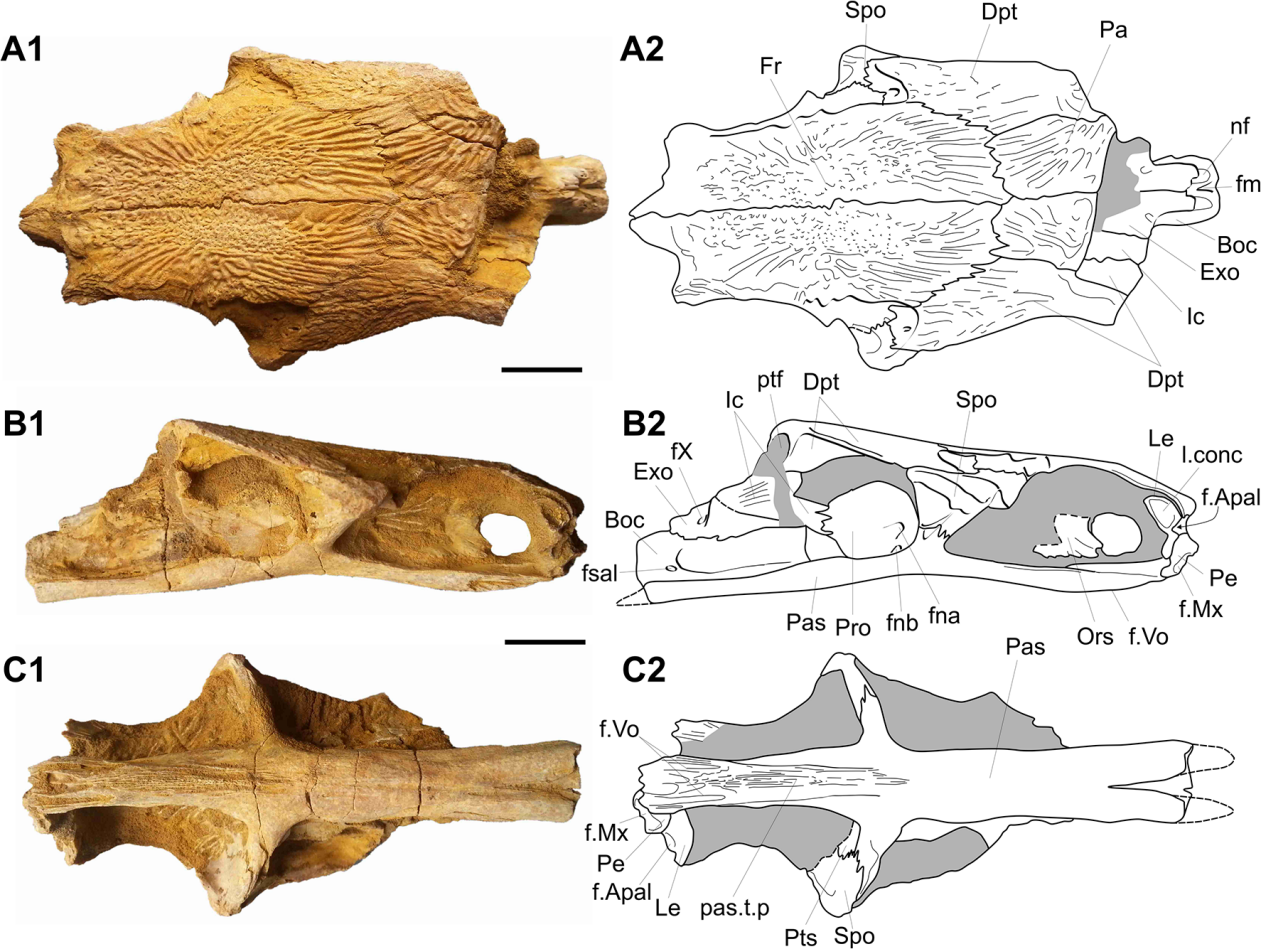
*Calamopleurus africanus* (MDE F-57). Photographs (1) and interpretative drawing (2) of the braincase in dorsal (A), right lateral (B) and ventral views. Boc, basioccipital; Dpt, dermopterotic; Exo, exoccipital; f.Apal, articular facet for the autopalatine; fm, foramen magnum; f.Mx, articular facet for the anterior process of the maxilla; fna, foramen for the hyomandibular trunk of the VII nerve, lateral line nerve and jugular canal; fnb, foramen for the orbital artery; Fr, frontal; fsal, lateral foramen in basioccipital; f.Vo, facets for the suture with the vomers; fX, foramen for the tenth nerve (vagal); Ic, intercalar; l.conc, lateral concavity; Le, lateral ethmoid; nf, neural facet for the first neural arch; Ors, orbitosphenoid; Pa, parietal; Pas, parasphenoid; pas.t.p, parasphenoid tooth plate; Pe, preethmoid; Pro, prootic; ptf, posttemporal fossa; Pts, pterosphenoid; Spo, autosphenotic;

MDE F-57 (Fig 8) is a well preserved and undistorted braincase found in an unknown locality of the Ifezouane Fm. In this description, we focus on characters that support the assignation of this specimen to *C*. *africanus*, and on characters not visible on the type material, but which differ from the type species (one of the rare extinct amiids, whose braincase is well known [52]). From the diagnosis of *C*. *africanus* three characters are features attached to the gular plate, one is from the upper jaw and one is from the frontal bone, the latter only being visible on the material described here. The frontal is relatively narrow, with the maximum width equal to 25% of the length (versus a width/length ratio of 30–33% for *C*. *cylindricus*). Although not specified in the diagnosis of *C*. *africanus*, this character concerns the ornamented part of the frontal only. Otherwise, the braincase of *C*. *africanus* is very similar to the braincase of *C*. *cylindricus*. The only difference that we notice is the presence of an anteroventral extension of the orbitosphenoid in *C*. *africanus*, which seems to be absent in *C*. *cylindricus* (compare Fig 8B with [52]’s Fig 304). The right ethmoid region of MDE F-57 is well-preserved, and shows differences with both *C*. *cylindricus* and *Amia calva*. The depth of the snout is slightly higher than in *C*. *cylindricus*, and significantly higher than in *A*. *calva*, and the ethmoid bones are much more ossified than in the latter species. In particular, there is a contact between the lateral ethmoid and the preethmoid, while a gap is present between both bones in *A*. *calva*. A contact is also present in *C*. *cylindricus*, but both ossifications appear to be less-developed than in the Moroccan species. Anteroventrally is an articular facet for the autopalatine. The lateral side of the lateral ethmoid is dug by an ovoid concavity, which is absent in *A*. *calva* and apparently also in *C*. *cylindricus*, and not visible on the type specimen of *C*. *africanus*. This concavity may have accommodated the anterior extremity of the anterior most supraorbital, a rather large ossification in *C*. *africanus* but absent in *A*. *calva* [51]. The preethmoid is proportionally large, strongly sutured to the parasphenoid ventrally and to the lateral ethmoid dorsally, and bears anteriorly a broad subrectangular facet, which forms the posterior wall of the socket into which fitted the anterior articular process of the maxilla, as in *A*. *calva* [52].

The snout of *C*. *cylindricus* is steeper than in *A*. *calva*, and we can predict that the snout of *C*. *african*us is still more abrupt based on the arrangement of its ethmoid region.

Teleostei Müller, 1846

Teleostei i.s.

*Erfoudichthys* Pittet, Cavin, Poyato-Ariza, 2010

*Erfoudichthys rosae* Pittet, Cavin, Poyato-Ariza, 2010

#### Remark

This species is based on an isolated head from an indeterminate locality of the Ifezouane Fm. [53]. Its phylogenetic position is unclear and it was regarded as either a stem Chanidae or as located outside the Ostariophysi [53].

#### Description of new material

**Fig 9:**
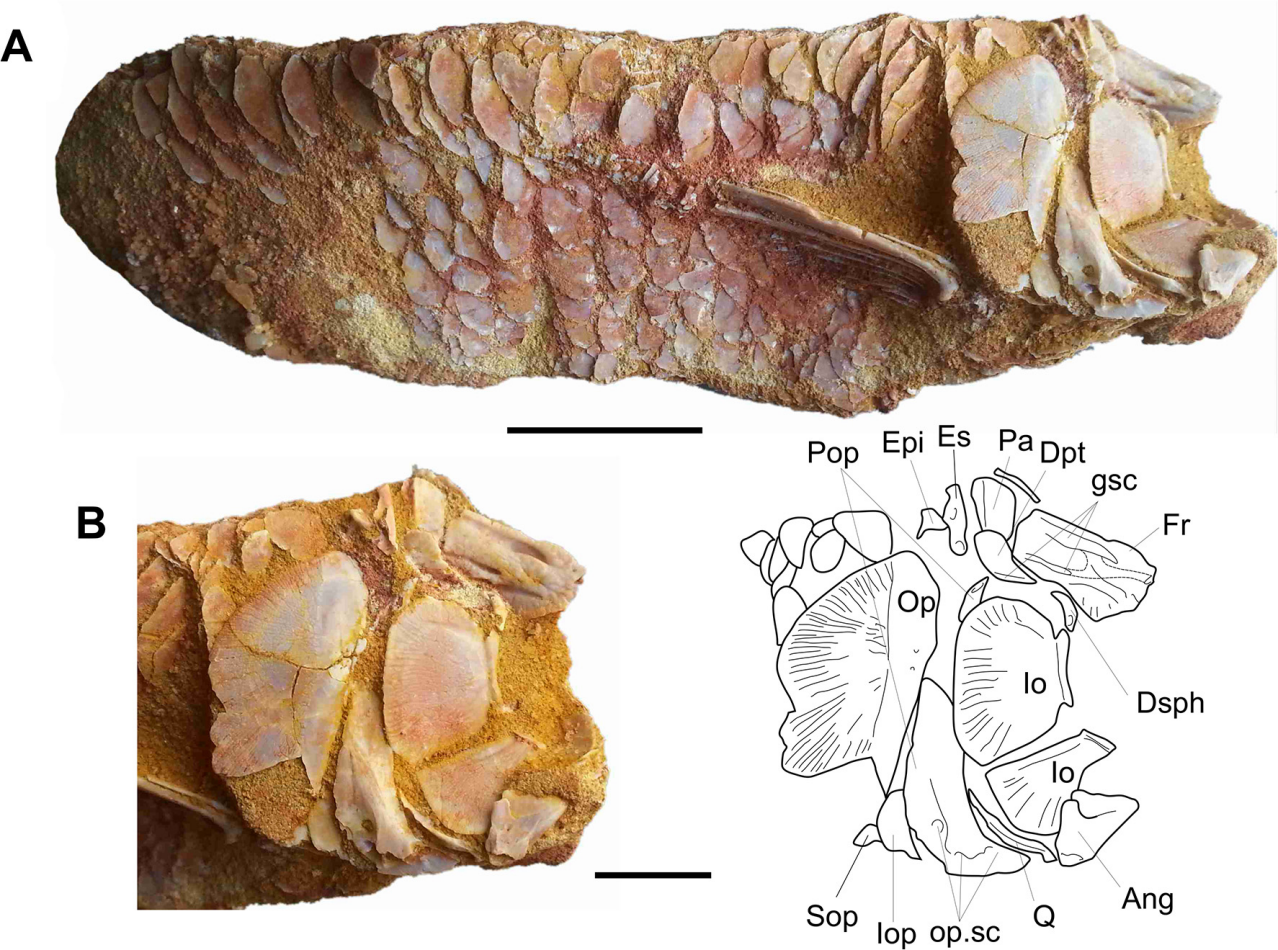
*Erfoudichthys rosae* (MDE F-56). A, right lateral view of the subcomplete specimen. B, photograph and drawing of the head in right lateral view. Ang, angular; Dpt, dermopterotic; Dsph, dermosphenotic; Es, extrascapular; Fr, frontal; g.s.c, groove for the sensory canals; Io, infraorbital; Iop, interopercle; Op, opercle; op.sc, openings of the sensory canal; Pa, parietal; Pop, preopercle; Q, quadrate; Sop, subopercle. Scale bars: A, 20 mm; B, 10 mm.

MDE F-56 is a portion of a body with the posterior part of the head of a small fish (Fig 9). The specimen is referred to *Erfoudichthys rosae* because of the ovoid and deep opercle, the pattern of the sensory canal in the preopercle, and the very large posteroventral and posterior infraorbital. The right frontal is well exposed and shows the beginning of a lateral extension above the orbit, which was not preserved on the type material. The otic sensory canal ran in a groove located along the posterior part of the lateral margin of the bone and extends anteriorly in a canal that curves medially. Based on the type material, the sensory canal is located in a groove in the anterior most portion on the frontal. Along the medial margin of the posterior part of the frontal is another groove, which likely connected the supraorbital sensory canal through a bone-enclosed tube. The dermopterotic is incompletely preserved, but it shows along its lateral margin a deep groove that probably accommodated the posterior part of the otic canal. Although the pattern of the sensory canals is difficult to reconstruct precisely, it is reminiscent of the situation found in some osteoglossomorphs.

The new material brings new information about this taxon. It was suggested that two infraorbitals were located posteriorly to the orbit, but this reconstruction probably misinterpreted a crack as a suture ([53]’s Figs 8.2 and 8.5). A single large posterior infraorbital is present in MDE F-56. Other new characters are the occurrence of pectoral fins with at least 10 rays, the first one being very long and broader than the other ones, and scales which are large, deeper than long and ornamented with a reticulated pattern but without visible circuli or furrows.

Although the previous phylogenetic analysis provided no clear results, some osteological features appeared to be reminiscent of ostariophysans, possibly gonorynchiforms. This possibility is not rejected here, but we notice also characters which may indicate relationships with osteoglossomorphs, in particular in the skull roof sensory canal system. A more detailed study of this taxon is pending.

Ichthyodectiformes Bardack & Sprinkle, 1969

Cladocyclidae Maisey, 1991 (sensu Cavin et al., 2012)

*Aidachar* Nesov, 1981

*Aidachar pankowskii* (Forey & Cavin, 2007)

#### Remark

In 2007, Forey & Cavin [54] described ‘*Cladocyclus’ pankowskii* on the basis of an isolated braincase from an unknown site of the Ifezouane Fm. Mkhitaryan and Averianov [55] revised the material of *Aidachar paludalis*, from the Turonian of Uzbekistan and originally regarded as a pterosaur by Nesov [56]. They found, in a cladistics analysis, that ‘*C’ pankowskii* should be placed in the genus *Aidachar*, which is resolved in a polytomy with *Cladocyclus* and the more derived ichthyodectiforms. This relationship, in particular the close affinity of *Aidachar* with *Cladocyclus*, supports the inclusion of both genera in cladocyclids according to the phylogenetic study of Cavin et al. [57], although further analyses will be necessary to test this hypothesis.

#### Description of new material

**Fig 10:**
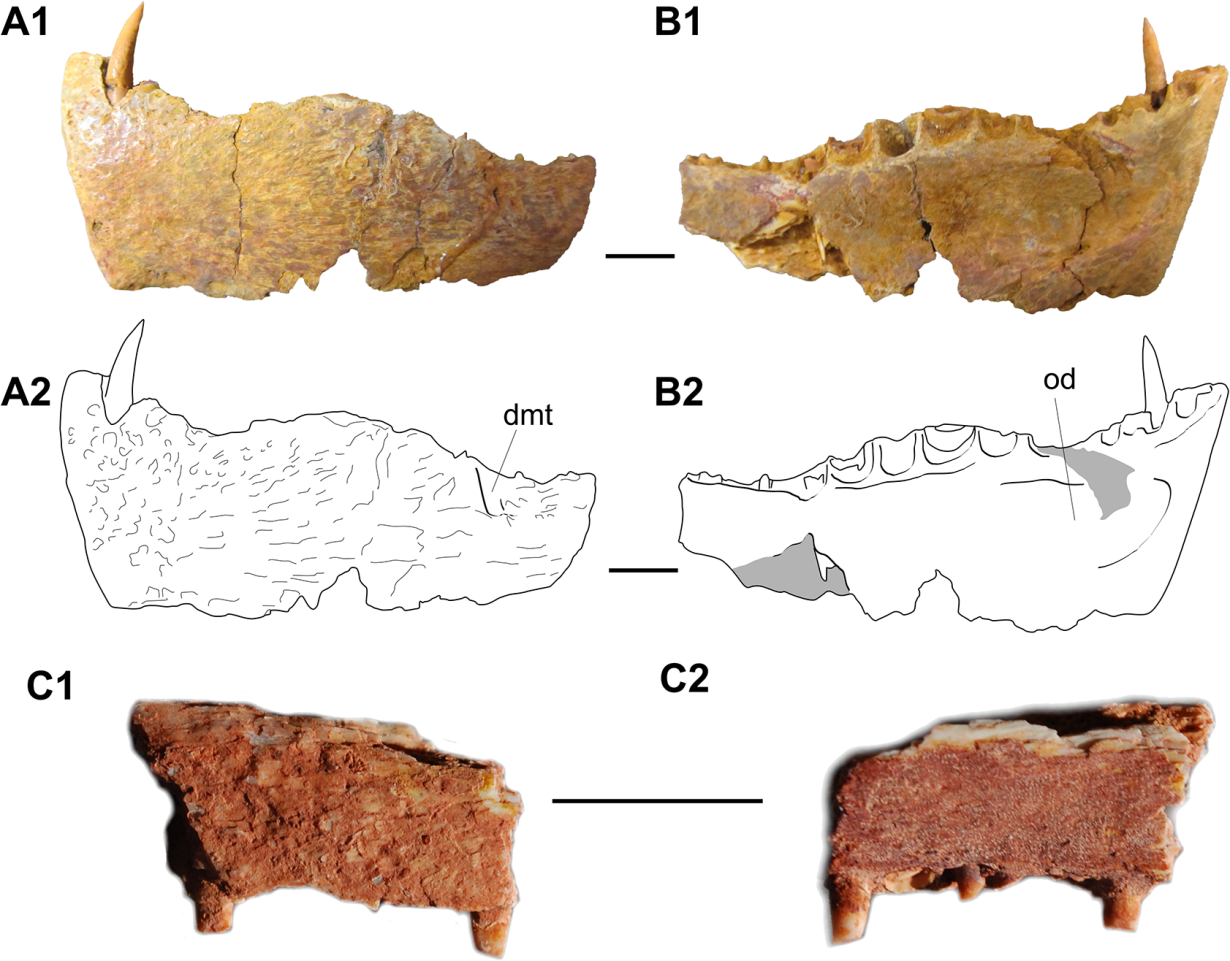
*Aidachar pankowskii*. A-B, right dentary (UMI-159). 1, photographs and 2, line drawings in lateral (A) and medial (B) views. dmt, depression for maxillary teeth; od, oval depression. C, maxilla? in lateral (1) and medial (2) view. Scale bars: 10 mm.

UMI-159 is a fragmentary dentary from an unknown locality of the Ifezouane Fm. with a single complete pointed tooth plus the alveoli, sometimes with fragmentary teeth, of 13 more teeth (Fig 10A and 10B, UMI-159). The ossification shares with the dentary of *A*. *paludalis* several characters, such as a sinusoidal alveolar border, the occurrence on the labial side of some depressions, very faint in the Moroccan specimen, probably for the enlarged maxillary teeth, a very deep and robust symphysis, large teeth, the largest ones concentrated on the convexity of the alveolar margin and an oval depression situated just posterior to the symphysis on the medial side. The latter character is also present in saurocephalids and *Gillicus*. In these fishes, the depression probably housed a well-developed anterior intermandibularis muscle. The similarities between the dentary described here and the dentary of *A*. *paludalis* allow referring this material to *A*. *pankowskii*.

A small fragment of jaw, found in Chaaft (level 1) in 2012 (Fig 10C, UMI-6), is referred with caution to this taxon. The bases of 6 longitudinally flattened teeth are preserved within deep sockets. Because the oral margin is straight and because no marked differences in size of the teeth are discernible, we refer it to a maxilla, keeping in mind that the fragmentary nature of the specimens makes the anatomical and systematic identifications uncertain.

Osteoglossomorpha Greenwood et al., 1966

Osteoglossiformes Regan, 1909

Notopteridae Bleeker, 1851

*Palaeonotopterus* Forey, 1997

*Palaeonotopterus greenwoodi* Forey, 1997

#### Remark

This species was defined by Forey in 1997 [58] on the basis of an isolated neurocranium from an unknown locality from the Ifezouane Fm. *P*. *greenwoodi* was included in the notopterids on the basis of a single synapomorphy: the occurrence of a supraorbital branch of the otic sensory canal, which runs parallel and medially to the supraorbital canal. Taverne & Maisey [59] described a second neurocranium, slightly more complete, and confirmed the inclusion in the notopterids thanks to a second synapomorphy associated with the organisation of the trigeminofascialis region. Later similar braincase material was found associated with the parasphenoid (missing in the formerly described specimens) which supported a large oval tooth plate [60,61]. This new material supports the inclusion of *Palaeonotopterus* in notopterids on the basis of three synapomorphies (but not anymore by the presence of a supraorbital branch of the otic canal, a character which was observed as also present in basal mormyrids), and one homoplasy. Based on one of these studies, the sister-group relationship with Mormyroidea (*Gymnarchus* + Mormyridae) is almost equally supported by three synapomorphies [61]. Interestingly Hilton [62], in his phylogenetic study of osteoglossomorphs, resolved a sister-group relationship of *Palaeonotopterus* with the mormyrids on the basis of 12 characters, although only two are known in *Palaeonotopterus* (parasphenoid sutures with the sphenotic and cleithrum with a broad medial lamina). Similarly, Wilson & Murray [63] found the relationships *Palaeonotopterus* + Mormyroidea on the basis of the same characters as those found by Hilton [62].

*P*. *greenwoodi* was regarded as co-specific [60] or closely related [61] to *Plethodus libycus*, known by a parasphenoid from the Cenomanian of Bahariya [9]. Schaal [32] described another species of *Plethodus* from Bahariya, *P*. *tibniensis*, on the basis of a lower tooth plate approximately pentagonal in shape.

#### Description of new material

**Fig 11:**
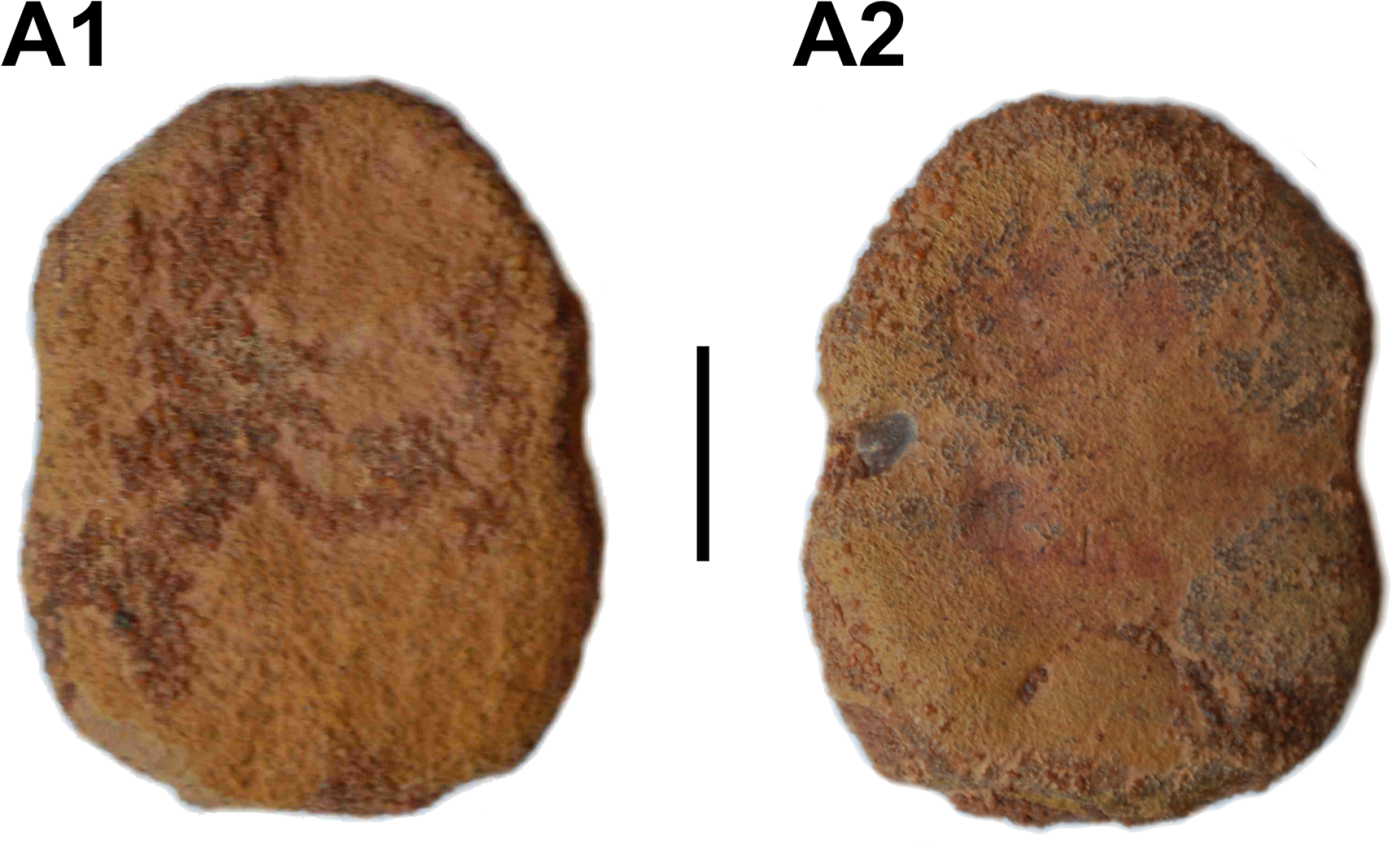
*Palaeonotopterus greenwoodi*. A, ventral tooth plate (UMI-10) in dorsal (1) and ventral (2) views. Scale bar: 10 mm.

Lower tooth plates were found in some localities. A complete one is oval in shape with a weak constriction in its mid-length (Fig 11, UMI-10). It is more reminiscent to the ventral tooth plate described by Taverne [60], although the latter does not show the constriction, than to the ventral tooth plate of *Plethodus tibniensis* described by Schaal[32]. More work is necessary to decipher the taxonomic issue between *Palaeonotopterus*, an osteoglossomorph, and the non-related plethodids, which are tselfatiiforms [64].

According to Taverne [60], the ventral tooth plate of *Palaeonotopterus* corresponds to the fusion of the dermobasihyal, dermobasibranchial, basihyal, basibranchial and hypobranchials of the first two branchial arches. The latter are still discernable as oblique strong crests on the ventral side of the plate.

Tselfatiiformes Nelson, 2006

Plethodidae Loomis, 1900

*Concavotectum* Cavin & Forey, 2008

*Concavotectum moroccensis* Cavin & Forey, 2008

#### Remark

This species was diagnosed on a large complete skull from an indeterminate locality from the Ifezouane Fm. Cavin & Forey [65] observed that *Concavotectum* is a Tselfatiiformes close to *Bachea* and *Enischnorhynchus*, from the Turonian of Colombia and from the Late Santonian – Early Campanian of Texas, respectively, but also very similar, and possibly conspecific with *Paranogmius* from Bahariya in Egypt, whose original material is now destroyed. In a review of the tselfatiiforms, Taverne & Gayet [64] placed *Bachea* and *Enischnorhynchus* in a derived position among the plethodids, a family of tselfatiiforms, while *Paranogmius* was regarded as the basal-most representative of the family because this genus retains a subtemporal fossa. A discussion of the intrarelationships among tselfatiiforms is beyond the scope of this paper, but we consider here *Concavotectum* (“*Paranogmius*”), and probably also *Bachea* and *Enischnorhynchus* as probable basal tselfatiiforms.

#### Description of new material

**Fig 12:**
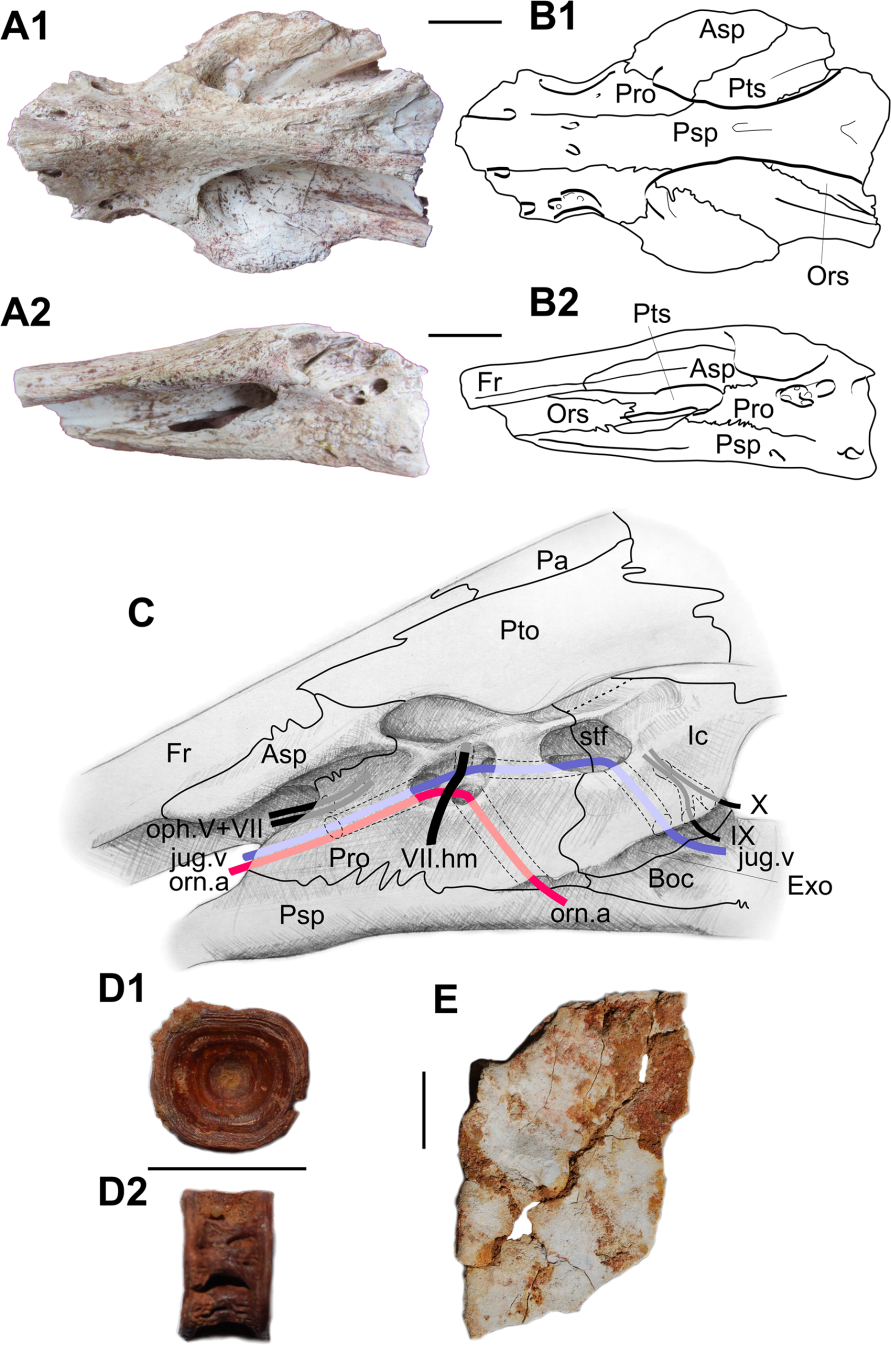
*Concavotectum moroccensis*. A-B, portion of a braincase (SESNE 1-52-2008). A, photographs and B, line drawings in ventral (1) and left lateral (2) views. C, tentative reconstruction of the posterior part of the braincase with the paths of some blood vessels and nerves in left lateral view. D, vertebra in anterior or posterior (1) and lateral (2) views (UMI-15). E, left opercle in lateral view (SESNE 1-48-2008). Asp, autosphenotic; Boc, basioccipital; Exo, exoccipital; Fr, frontal; Ic, intercalar; jug.v, jugular vein; oph.V+VII, ophthalmic ramic of the trigeminal and facial nerves; orn.a, orbitonasal artery; Ors, orbitosphenoid; Pa, parietal; Pro, prootic; Psp, parasphenoid; Pto, pterotic; Pts, pterosphenoid; stf, subtemporal fossa; VII.hm, hyomandibular branch of the trigeminal nerve; IX, glossopharyngeal nerve; X, vagus nerve. Scale bars: 20 mm.

Braincases – Fragments of braincases of *Concavotectum* have been found in Tizi Moumrad in 2008 (Fig 12A and 12B, SESNE 1-52-2008, -1-53-2008, 1-54-2008). We mention here some characters that confirm uncertain features provided in Cavin & Forey [65] and complete the description of the species. As on the holotype specimen, several of the sutures between the dermal bones of the skull roof are obscured, probably because of the large size of the specimens. This is particularly the case for the posterior limit between both frontals. We confirm previous suggestion that the bone sutured ventrally to the autosphenotic is a pterosphenoid (SESNE 1-52-2008), although we cannot observe the unusual features of this ossification, i.e. its unpaired condition and its lining with the frontal, because the parasphenoid is preserved and prevents the observation of this part of the braincase. The identification of the pterosphenoid is comforted by the occurrence of an orbitosphenoid, not visible on the previously described material. The posterior part of this bone, only preserved, is triangular in ventral view and is tightly sutured to the pterosphenoid posteriorly, to the frontal dorsally, and to the parasphenoid ventrally. The bone expands laterally frontwards, following the broadening of the parasphenoid and of the ethmoid region, which is a feature characteristic of this taxon. We also confirm the proposed path for the jugular vein (SESNE 1-52-2008, -1-94-2008, 1-95-2008): anteriorly, from the orbital cavity, the vein entered the jugular canal in the prootic just beneath the opening for the superficial ophthalmic branches of the facial and trigeminal nerves (oph.V+VII) and exits in the anterior part of the elongated depression, in the bottom of which opens the canal for the hyomandibular trunk of the VII. The jugular vein crossed this depression, entered a canal dug between the posterior extremity of this depression and the subtemporal fossa, then crossed this fossa and entered another canal that runs in the exoccipital and opens posteriorly in the groove wedged between the intercalar and the exoccipital. Posterior to this pore, two others pierced the exoccipital (SESNE 1-53-2008, 1-54-2008) for the exits of the vagus and glossopharyngeal nerves. In a previous description of another specimen (BMNH P.66350), a single pore accommodated both nerves, indicating that this character is probably polymorphic in this species ([65]: Fig 8). In the bottom of the depression for the exit of hyomandibular trunk of the VII opens the canal for the orbitonasal artery, which runs ventrally and opens near the border between the prootic and the parasphenoid.

As mentioned by Cavin & Forey [65], the path of the jugular vein is unusual by being enclosed by bone for much of its cranial course (Fig 12C). This pattern is reminiscent of the ichthyodectiform pattern, in which a canal opens posteriorly in the exoccipital, then runs anteriorly and opens in the subtemporal fossa. In ichthyodectiforms the IX and X nerves follow the posterior part of the canal, then angle anteromedially and enter the endocranial cavity. In *Concavotectum*, the canals for the nerves are independent from the canal for the vein.

Postcranial remains—In most of the visited localities (Chaaft [level 1], Douira [levels 1 and 2], ANG [level A], ANG W [level C]), isolated vertebral centra are referred with caution to *C*. *moroccensis* (Fig 12D). They are deeper than long, and ornamented on their lateral sides with 1 to 4 deep grooves, and with deep pits dorsally for accommodating the neural arches and ventrally for parapophyses and haemal arches. An isolated base of a fin ray with a complex articular facet was found in Douira 2 (Temp -28). It is tentatively referred to *C*. *moroccensis* by comparison with the first ray of the pectoral fin of the holotype. The ray of the holotype being still in articulation, its internal face is not visible and this identification is only tentative. An isolated opercle, deeper than long with a tapering ventral extremity, is referred to *C*. *moroccensis* by comparison with the holotype (Fig 12E).

### Diets and trophic web

#### Lungfishes

Because the tooth plate morphology of Cretaceous lungfishes is very similar to those of recent taxa, we assume that their diet were similar. The Australian lungfish *Neoceratodus forsteri* is an opportunistic omnivore, with the adults (with complete tooth plates as opposed to the isolated conical tooth cups of juveniles) feeding on plant material, invertebrates, as well as small vertebrates, such as tadpoles and small fishes [66]. The South American lungfish *Lepidosiren paradoxa* has a rather similar omnivorous diet [67], and the four African *Protopterus* species are also opportunistic [67]. Trophic level of living lungfishes varies between 3.1 (*Lepidosiren*) and 3.3 (*Neoceratodus*), and we keep here an average value of 3.2 for the three taxa of the Kem Kem assemblage.

Large tooth plates of *Neoceratodus* are present in the Ifezouane Formation, but not described here. The one figured in Fig 2C corresponds, when compared to a modern *N*. *forsteri*, to an individual of ca 700 mm of total length. We do not know the general morphologies of *Arganodus* and *Ceratodus*. If we use the same proportion than for *Neoceratodus*, tooth plates of *C*. *humei* and *A*. *tiguidiensis* correspond to individuals of ca 90 mm in total length.

#### Mawsoniid coelacanths

We have no direct evidence of the diet of mawsoniid coelacanths. The extant *Latimeria* is a carnivorous fish, which catch its preys probably by suction [30]. Because the skull morphology is relatively conservative and because the cranial kinesis of Mesozoic coelacanths is rather similar to the cranial kinesis of *Latimeria*, we hypothesise that the edentulous mawsoniid also sucked their prey items, which might have been large according to the large size of some of these fishes. We use the tropic level of 4.4 identified for the living *Latimera chalumnae*. If the body proportions of *Mawsonia brasiliensis* are applied to the isolated dentary from the Kem Kem, the obtained total length of the fish is circa 3.5 meters. This value is used here for *M*. *lavocati*, although it may correspond to cf. *Axelrodichthys*. The specimen of Mawsoniid indet. figured in Cavin & Forey ([27]: 496) corresponds to an individual of 765 mm based on the same comparison with *M*. *brasiliensis*. This value is retained here for cf. *Axelrodichthys*, keeping in mind the comment above.

Giant coelacanths as being filter-feeders were suggested by Schwimmer [68] for *Megalocoelacanthus* and by Maisey [12] for *Mawsonia* and *Axelrodichthys*, but further researches are necessary to prove this hypothesis.

#### Polypterids

Articulated polypterids have been found in the Djbel Oum Tkout locality [69] located in the Aoufous Fm., which has yielded a different assemblage corresponding to a different palaeoenvironment [1,2]. Only isolated fragmentary remains have been found in the detritic beds of the Ifezouane Fm. so far, indicating that caution should be taken when we address the diet of these fishes. However, the resemblance of the dentition and of the preserved maxilla with living polypterids indicates that they likely had a similar diet. Recent polypterids are nocturnal predators that feed on fishes, amphibians and aquatic invertebrates. We suspect that the polypterids from the Kem Kem had a similar diet, with a preference for large preys for cf. *Bawitius*. Living polypterid species have a trophic level between 2.6 and 3.7 and we retain here a value of 3 for the fossil taxa. The sizes of the bony remains and of the scales are comparable to the material from Bahariya referred to *Bawitius*. Grandstaff et al. [34] suggest a total size of 2.5 to 3 meters of total length. We keep the lowest value for cf. *Bawitius* here. We have no indication for estimating the body size of *Sudania* and *Bartschichthys*.

#### Ginglymodians

?*Lepidotes pankowskii* has a pavement of small crushing teeth of its vomers, indicating a diet comparable to the diet of lungfish. We assign it a trophic level of 3.2. The body size of the holotype of this species was estimated to 160 cm [40], but observation (LC) of isolated scales measuring ca 10 cm in total length with a ganoid exposed surface of ca 4 cm in length, indicates specimens of ca 250 cm in total length.

The jaws are not visible on our available material of obaichthyids and we discuss the possible diet of these fishes on the basis of the sister-species from the Santana Formation described by Grande [43]. Obaichthyids have a very specialised jaw apparatus, with a rostral region extending anteriorly beyond the lower jaw symphysis, with teeth (without plicidentine) concentrated to the anterior part of the mandible, a toothed lateral process of the premaxilla, and a small gape [43]. This arrangement indicates a very different mode of feeding than the living piscivorous gars and is evidence that obaichthyids did not feed on large prey items. We rather suggest that they fed on invertebrates and gave them a trophic level of 3 corresponding to this diet in other groups. Obaichthyids from the Santana Formation are ca 600 mm in length, but the subcomplete individual of *O*. *africanus* indicates a smaller size, ca 300 mm. However, some large (28 mm in height) isolated scales of *O*. *africanus* (e.g., [16]: pl. XI, Fig 8) clearly show that *O*. *africanus* could reach a much larger size. We cannot estimate the length for *D*.? *kemkemensis*.

*Oniichthys falipoui* is very close to the living gar *Atractosteus* (cogeneric according to Grande [43]) and consequently had likely a similar diet. The three living species feed mostly on fishes, but also birds, turtles and small mammals for *A*. *spatula*. Because *O*. *falipoui* is comparatively small in size, we suggest that it fed mostly on fishes and possibly invertebrates. We use the trophic level of the living species, which is 4. Based on the three available subcomplete specimens, we estimate the body length of this species to ca. 400 mm.

The opisthocoelous abdominal centrum of an indeterminate Lepisosteoidea figured in Fig 5F indicates the occurrence of much larger individuals of this clade, circa 130 cm in standard length if we applied body proportion of recent gars.

#### Halecomorphs

The only living amiid, the North American bowfin (*Amia calva*) is a voracious and opportunistic fish, which feeds on other fishes, frogs, crayfish, insects and shrimps with a trophic level of 3.8. Specimens of *Calamopleurus cylindricus* from the Santana Formation in Brazil are regularly found with prey items, mostly *Vinctifer* sometimes of proportionally large size [12], indicating that *Calamopleurus* was likely more ichthyophagous than the recent *Amia*. We give a trophic level of 4 to *Calamopleurus africanus*. Based on a comparison with *C*. *cylindricus*, the holotype specimen of *C*. *africanus* had a total length of ca. 500 mm.

#### Ichthyodectiforms

The general body morphology, gut contents, and the jaw anatomy and biomechanics make little doubt that Cretaceous ichthyodectiforms were voracious predators, with the possible exception of the genus *Gillicus* [57]. Standard length and mouth size are proportionally related to prey size in piscivorous fishes [70] and we suspect that *Aidachar pankowskii* fed on proportionally large preys. Direct evidence of piscivory (gut content) was observed in the closely related *Cladocyclus* from the Santana Formation [12]. We consider a trophic level of 4.2 for *A*. *pankowskii*, which is in the range of the trophic level of the *Sphyraena* species. Based on comparison with complete specimens of *Cladocyclus* from the Santana Formation, the fragmentary dentary (Fig 10A and 10B) corresponds to an individual about 1 meter in total length.

#### Osteoglossomorphs

**Fig 13:**
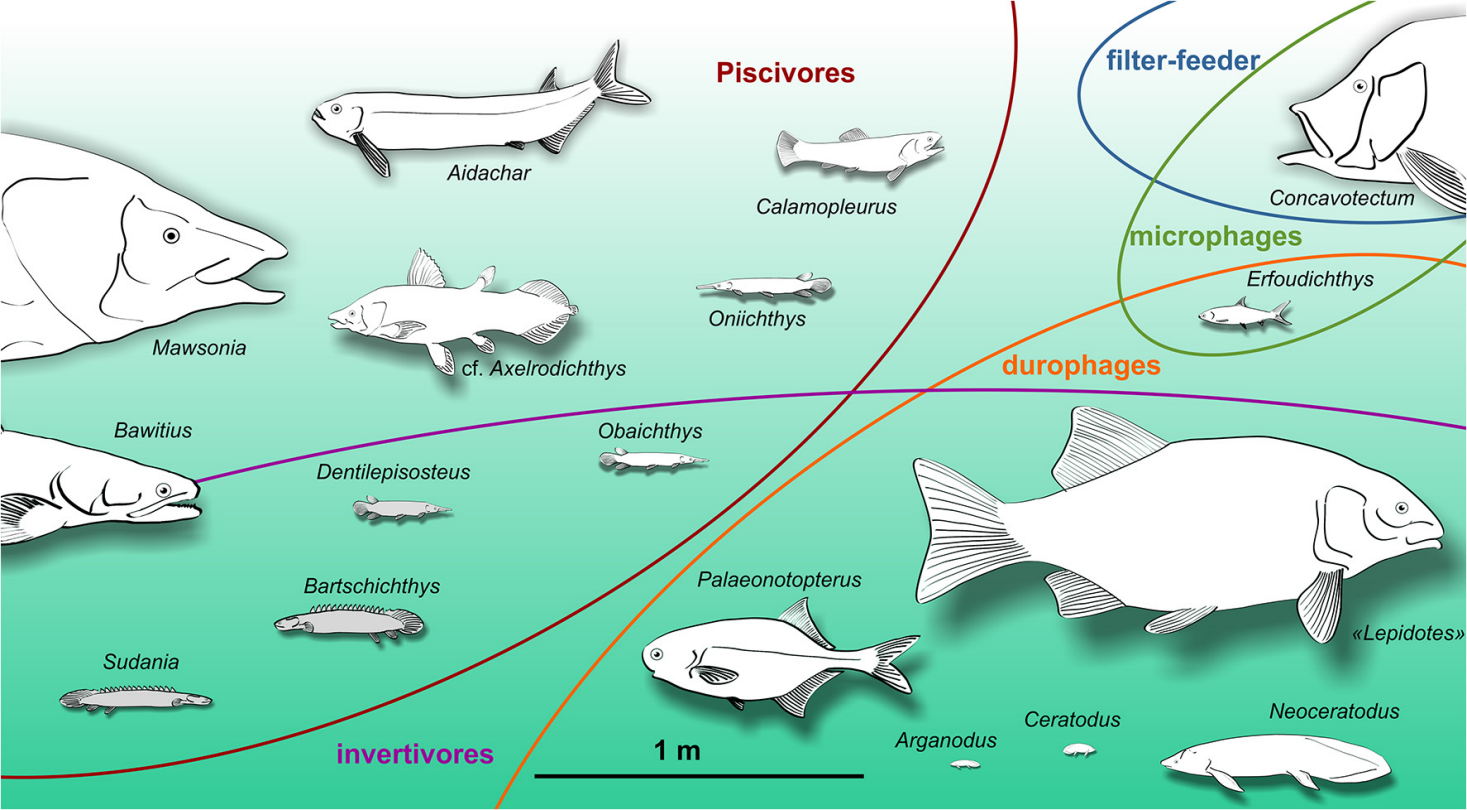
Bony fish assemblage of the Kem Kem beds and trophic niches figured at scale. Grey figures indicate taxa with uncertain body size.

Taverne [71] described a hyomandibula of *Palaeonotopterus greenwoodi*, which differs considerably from hyomandibulars of Recent notopterids, mormyrids and gymnarchids, probably in connexion with the highly specialized palato-lingual bite. A primary bite between parasphenoid and tongue is a feature of ossteoglossomorphs, which correspond to a mix of several distinct characters [62]. In *Palaeonotopterus*, however, this apparatus is highly specialized because of the broad and thick lower and upper tooth plates. Meunier et al. [72] showed that the lower tooth plate is formed by several layers of highly packed teeth, with the crushing surface formed by the worn apex of the last layer of teeth. The morphology and histology of this crushing buccal apparatus, comparable with tetraodontid jaws, are indicative of a durophagous diet [72]. We give to this species a trophic level of 3.2 by comparison with tetraodontids, which have trophic levels mostly ranging from 3 to 3.5. The reconstruction of the body shape of *P*. *greenwoodi* varies greatly according to the model we used, i.e. either *Notopterus* or *Petrocephalus* (a basal mormyrid, which is used for the reconstruction in Fig 13), but the total length estimation is almost similar with both models, i.e. between 700 and 1000 mm.

#### Tsefatiiforms

Jaws are preserved on the holotype specimen. They are edentulous and associated with a funnel-like mouth, a deep branchial chamber and long gill rakers, which support that this fish was a probable filter feeder [62]. We assign the average trophic level of the menhaden (*Brevoortia*), a marine clupeid filter feeder, to estimate the trophic level of *Concavotectum moroccensis* (2.5). Based on the holotype, an isolated skull, the total length is estimated to reach 1.5 meter if proportion of *Tselfatia* is used.

#### Teleosts indeterminate

Based on its mouth morphology, without marginal teeth and a small gape, *Erfoudichthys rosae* was probably very low in the trophic level. We use the trophic level of *Chanos*, 2, for this species. Based on the proportion of *Chanos*, the total size of *E*. *rosae* was ca. 45 cm.

**Fig 14:**
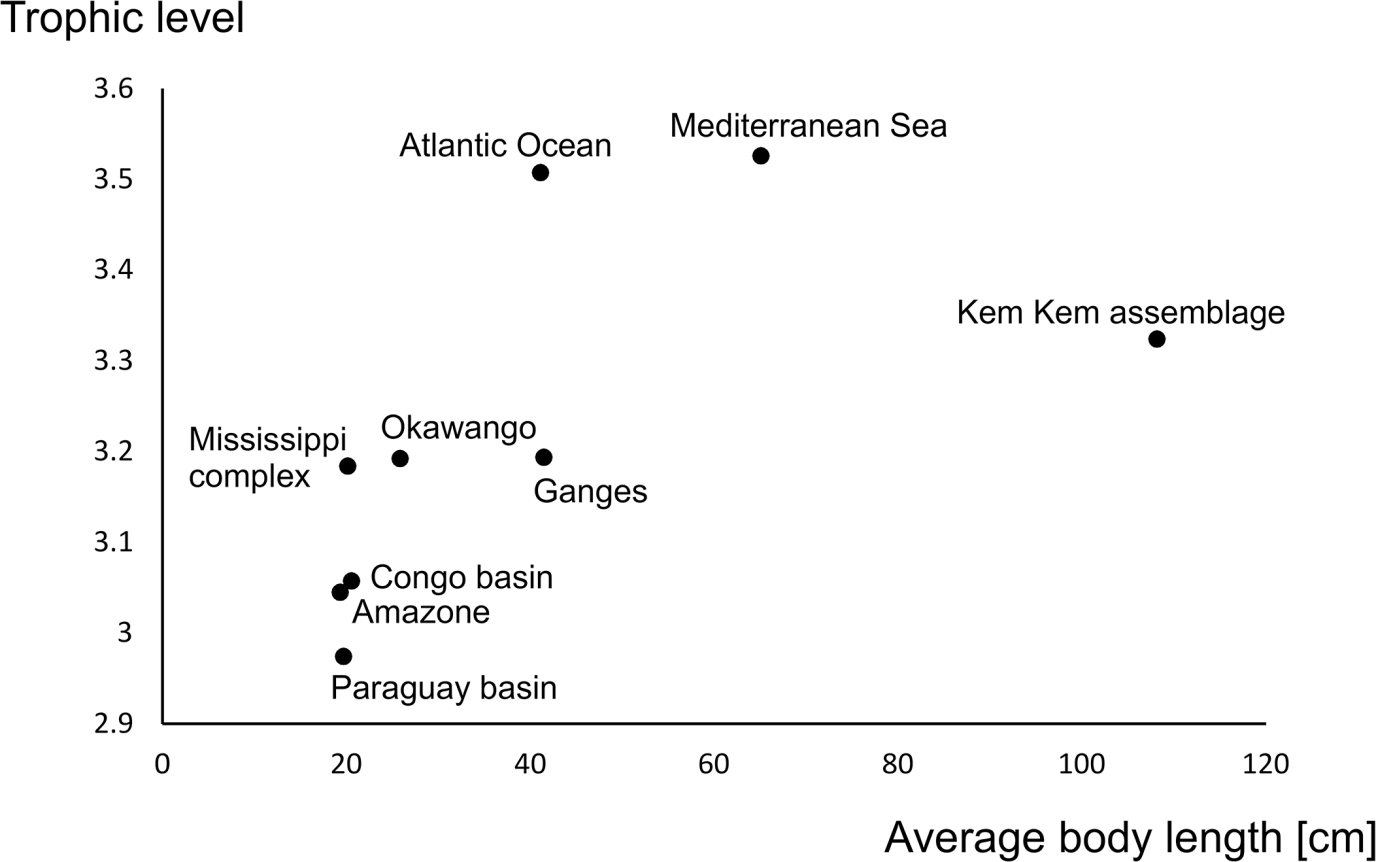
Average trophic levels against average body size of various recent marine and freshwater assemblages calculated from data collected in Fishbase, plus the Kem Kem beds assemblage.

Fig 13 and Table 1 show the trophic position (or level) and body size of the Kem Kem taxa. Fig 14 plots the average body size against the average trophic levels for several recent bony fish assemblages on the basis of data from Fishbase (S1 Table), with comparable value for the Kem Kem assemblage as estimated above.

**Table 1:** Taxa of the Kem Kem assemblage, with their reconstructed body length and trophic level.

	Total length (mm)	Trophic level
*Ceratodus humei*	90	3.2
*Arganodus tiguidiensis*	90	3.2
*Neoceratodus africanus*	700	3.2
*Mawsonia lavocati*	3500	4.4
cf. *Axelrodichthys*	765	4.4
cf. *Bawitius*	2500	3
*Bartschichthys* sp.		3
*Sudania* sp.		3
?*Lepidotes pankowskii*	2500	3.2
*Obaichthys africanus*	300	3
?*Dentilepisosteus kemkemensis*		3
*Oniichthys falipoui*	400	4
*Calamopleurus africanus*	500	4
*Aidachar pankowskii*	1000	4.2
*Palaeonotopterus greenwoodi*	850	3.2
*Concavotectum moroccensis*	1500	2.5
*Erfoudichthys rosae*	450	2

## Discussion

Although some of the bony fish taxa described above are represented by complete or subcomplete specimens, most of the available data rests on isolated elements. Most of these fragmentary fossils were found in the bottom of palaeochannels, together with small pebbles, indicating an aquatic environment with relatively high energy. These environmental parameters may have caused a sorting of the organic remains with a preferably preservation of highly mineralized elements, such as teeth and ganoid scales. This bias may have affected the general picture of the taxonomic composition of the fish assemblage. Keeping this reservation in mind, the taxonomic composition of the bony fish assemblage shows peculiar features when compared with other Cenomanian bony fish assemblages. Sarcopterygian taxa represent a large part of the taxic composition of the assemblage (29.5%). Among ray-finned fishes, cladistians represent an unusual high proportion with three genera (17.6%), although their occurrence is pending to be proven. Holosteans represent a still higher part of the assemblage (29.5%), with a higher proportion of ginglymodians (23.5%) than halecomorphs (5.9%). Teleosts are rare in the Kem Kem assemblage (23.5%) when compared to coeval marine assemblages, in which they are dominant.

The most comparable bony fish assemblages are the assemblage from the Early Cenomanian of Bahariya, Egypt, which probably represents the same palaeoecosystem [73], and the bony fish assemblage from the Santana Formation, Brazil. In the latter, 24 genera are present [74]: sarcopterygians (8.3%), ginglymodians (16.7%) and halecomorphs (12.5%) represent significant parts of the assemblage, but cladistians are apparently absent. The proportion of teleosts in the Santana Formation is more than twice the proportion in the Kem Kem assemblage (62.5% versus 23.5%). The Santana Formation assemblage is possibly slightly older than the Kem Kem beds assemblage, probably Late Albian in age [75]. The taxonomic affinities between both assemblages is due to palaeogeographical affinities of both faunas, and probably to a relatively similar palaeoenvironment, i.e. a closed marine environment with continental inputs for the Santana Fm. and continental environment with brackish elements for the Kem Kem beds.

The trophic web of the Santana Fm. assemblage [12] was well-established on the basis of direct evidence, and a preliminary reconstruction of the trophic web of the Moroccan Turonian marine locality of Goulmima was proposed on the basis of direct and indirect evidence [15]. The three assemblages (Santana, Kem Kem and Goulmima) show similar characteristics: 1) Top predators are ichthyodectiforms and amiids, with the amiid *Calamopleurus*, the cladocyclids *Ghrisichthys* and *Aidachar* as the largest topmost predators in the Santana, Goulmima and Kem Kem assemblages, respectively; 2) There are several levels of piscivores, four in the Santana assemblage with *Calamopleurus* / *Cladocyclus* / *Rhacolepis* / *Santanichthys* and at least three in the Goulmima assemblage, *Ghrisichthys* / *Goulmimichthys* / small *Enchodus* (this feature is unknown in the Kem Kem assemblage); 3) Durophagous feeders are present in the three assemblages, but with different representatives: pycnodonts and albuliforms in the Goulmima and in the Santana assemblages,? *Lepidotes*, *Palaeonotopterus* and lungfishes in the Kem Kem assemblages; 4) Filter feeders are present in the Santana (*Vinctifer*) and in the Kem Kem (*Concavotectum*) assemblages. The latter point is worth noting: Maisey [12] compared *Vinctifer* with the recent *Polyodon* because both have a maxilla fixed against the neurocranium and no free premaxilla, to the contrary of teleostean filter feeders with a protrusible jaws. *Concavotectum*, although a teleost, also had secondary a non-protrusible jaw with fused premaxillaries probably adapted for filter-feeding. In the Santana assemblage, *Vinctifer* was the preferred prey of *Calamopleurus*, while *Concavotectum*, although apparently abundant in number, was too large to be a common prey.

For recent faunas, the average body size of the compared marine assemblages is slightly higher than the average body size of the selected freshwater assemblages. The distinction between both kinds of environments is more marked along the other axis, the average trophic level, which is markedly more elevated for the marine than for the freshwater assemblages. For the extinct Kem Kem assemblage, the average trophic level is intermediate between the recent freshwater and marine faunas, but the extinct assemblage differs mostly by the very high average body size of the taxa. One taxon probably exceeded 3 meters in length (*Mawsonia*), at least two taxa were between 2 and 3 meters in length (cf. *Bawitius* and? *Lepidotes*) and two were between 1 and 2 meters in length (*Aidachar*, *Concavotectum*). Moreover, evidence, not recorded here, indicates the occurrence of very large *Neoceratodus*, probably several meters in length.

It should be noticed that both the average body length and the average trophic level of the Kem Kem assemblage are probably significantly affected by the fragmentary nature of the preservation. Keeping this limitation in mind, it is worth mentioning that the Kem Kem fish assemblage shows two features that were initially proposed for the Kem Kem terrestrial assemblage, i.e. that it is composed of a high proportion of carnivorous taxa and of a high proportion of species represented by large specimens [76]. The ecological feature of the Kem Kem fish assemblage, which still needed to be confirmed by analysing more complete fossil samples, is a new support of the peculiar ecological nature of the vertebrate Kem Kem assemblage if compared with extant vertebrate ecosystems.

## Conclusion

The study of a new set of fish remains from the Kem Kem assemblage, mostly fragmentary, together with a critical examination of previously described material, reveals a moderately diversified assemblage of 17 bony fish species. The sarcopterygian component is important, with at least three lungfishes and two coelacanths, and the actinopterygian assemblage is characterised by the dominance of non-teleostean taxa, in particular cladistians and holosteans. Comparisons of the estimated average trophic level of the extinct fish assemblage with extant assemblages indicate a higher proportion of carnivorous taxa with high trophic level than in modern freshwater ecosystems, but lower than in marine ecosystems. The topmost trophic level in the Kem Kem aquatic vertebrate assemblage, however, was not occupied by fishes but by crocodiles and spinosaurs.

Several bony fish taxa in the Kem Kem assemblages are represented by large or giant individuals, in particular mawsoniid coelacanths, a polypterid, a ginglymodian, an osteoglossomorph and a tselfatiiform. Reasons that triggered fish species to reach such large size are unknown, but they might be rooted in abiotic environmental features that characterized this assemblage, such as its large spatial extension and the warm climate of that time [6,77].

## Supporting Information

S1 Text List of material examined. (DOCX)

S1 Table Data (body sizes and trophic levels) for fish assemblages. (XLSX)
