# Supplementary material for: Taxonomic Composition and Trophic Structure of the Continental Bony Fish Assemblage from the Early Late Cretaceous of Southeastern Morocco
Source: PLoS One. 2015 May 27;10(5):e0125786. doi: 10.1371/journal.pone.0125786 (PMC4446216; doi:10.1371/journal.pone.0125786)
Supplement: S1 Text — (DOCX) [file pone.0125786.s001.docx]

**Taxonomic composition and trophic structure of the continental bony fish assemblage from the early Late Cretaceous of southeastern Morocco**

**Lionel Cavin^1^, Larbi Boudad^2^, Haiyan Tong^3^, Emilie Läng^1^, Jérôme Tabouelle^4^, Romain Vullo^5^**

**1** Dpt. de Géologie et Paléontologie, Muséum de Genève, CP 6434, 1211 Genève 6, Switzerland, **2** Université Moulay Ismail, Faculté des Sciences, Département de Géologie BP. 11201, Zitoune, 50070 Meknès, Morocco, **3** Palaeontological Research and Education Centre, Mahasarakham University, Kantarawichai, Mahasarakham 44150, Thailand, **4** Jérôme Tabouelle La CREA- Fabrique des Savoirs- Musée d’Elbeuf, **5** Laboratoire Géosciences Rennes, UMR CNRS 6118, Université de Rennes 1, 263 avenue du Général Leclerc, 35042 Rennes, France

**Supporting Information S1**

**Material described in the article**

UMI-1: *Mawsoniid*, dentary, AN’G, 2012

UMI-2: *Arganodus tiguidiensis*, tooth plate, Chaaft 1, 2012

UMI-3: ‘*Neoceratodus’ africanus*, tooth plate, Chaaft 1, 2012

UMI-4: *Ceratodus humei*, tooth plate, Douira, 2012

UMI-5: ?‘*Neoceratodus’ africanus* , pterygoid, AN’G (B), 2012

UMI-6: *Aidachar pankowskii*, fragment of maxilla, Chaaft 1, 2012

UMI-7: ‘*Neoceratodus’ africanus*, tooth plate+pterygoid, Khetitila, 2013

UMI-8: ‘*Neoceratodus’ africanus*, angular, Chaaft 3, 2013

UMI-9: Lepisosteoidei indet., caudal centrum, Chaaft 1, 2012

UMI-10: cf *Palaeonotopterus*, tooth plate, AN’G W(A), 2012

UMI-11: *?Dentilepisosteus kemkemensis*, 3 isolated scales, Chaaft 1, 2012

UMI-12: cf. *Bawitius*, 2 isolated scales, Chaaft 1, 2012

UMI-13: Ginglymodi indet, 2 isolated scales, Chaaft 1, 2012

UMI-14: *Obaichthys africanus*, 1 isolated scale, Chaaft 1, 2012

UMI-15: ?*Concavotectum moroccensis*, 8 complete vertebral centra + fragments, Chaaft 1, 2012

UMI-16: ?Mawsonia, Pop or Sop?, Chaaft 1, 2012

UMI-17: *Obaichthys africanus*, 2 isolated scales, Douira 1, 2012

UMI-18: ?*Concavotectum moroccensis*, 7 complete vertebral centra, Douira 1, 2012

UMI-19: ?*Concavotectum moroccensis*, 2 complete vertebral centra and 1 incomplete, AN’G W (C), 2012

UMI-20: ‘*Neoceratodus’ africanus*, tooth plate, AN’G W (C) 1, 2012

UMI-21: cf. *Bawitius*, 1 isolated scale, AN’G W (C) 1, 2012

UMI-22: ‘*Neoceratodus’ africanus*, angular, AN’G W (C) 1, 2012

UMI-23: ?*Concavotectum moroccensis*, 7 complete and incomplete vertebral centra, Douira 2, 2012

UMI-24: ?*Concavotectum moroccensis*, base of a fin ray, Douira 2, 2012

UMI-25: *Palaeonotopterus*?, Fragment of tooth plate, AN’G, level (B), 2012

UMI-26: *Obaichthys africanus*, 1 isolated scale, Chaaft 3, 2012

UMI-27: ?*Concavotectum moroccensis*, ?supraoccipital, Douira 2, 2012

UMI-28: cf. *Bawitius*, 1 isolated scale, AN’G (A), 2012

UMI-29: ?*Concavotectum moroccensis*, 1 complete vertebral centrum, AN’G (A), 2012

UMI-30: cf. *Axelrodichthys*, right principal coronoid, locality unknown

UMI-31: *Obaichthys africanus*, subcomplete specimen, locality unknown.

UMI-159: *Aidachar pankowskii*, fragmentary dentary, locality unknown.

MDE-F13: *Oniichthys falipoui*, locality unknown

MDE-F56: *Erfoudichthys rosae*, locality unknown

MDE-F57: *Calamopleurus africanus*, locality unknown

MDE-F60: indeterminate Lepisosteoidea, abdominal centrum, locality unknown

SESNE 1-03-2008: *Bawitius* (= “*Stromerichthys*”, polypterid), maxilla, Tizi Moumrad, 2008

SESNE 1-48-2008: *Concavotectum moroccensis*, right opercle, Tizi Momrad, 2008 (1-48-2008)

SESNE 1-52-2008: *Concavotectum moroccensis*, braincase, Tizi Moumrad, 2008

SESNE 1-53-2008: *Concavotectum moroccensis*, braincase, Tizi Moumrad, 2008

SESNE 1-54-2008: *Concavotectum moroccensis*, braincase, Tizi Moumrad, 2008

SESNE 1-61-2008: *?Lepidotes pankowskii*, 2 isloated teeth, Tizi Momrad, 2008
